# Integrated bioinformatic analysis reveals the underlying molecular mechanism of and potential drugs for pulmonary arterial hypertension

**DOI:** 10.18632/aging.203040

**Published:** 2021-05-18

**Authors:** Haoru Dong, Xiuchun Li, Mengsi Cai, Chi Zhang, Weiqi Mao, Ying Wang, Qian Xu, Mayun Chen, Liangxing Wang, Xiaoying Huang

**Affiliations:** 1 Division of Pulmonary Medicine, The First Affiliated Hospital of Wenzhou Medical University, Key Laboratory of Heart and Lung, Wenzhou 325000, Zhejiang, P.R. China; 2 The First Clinical Medical College, Wenzhou Medical University, Wenzhou 325000, Zhejiang, P.R. China

**Keywords:** PAH, DEGs, hub gene, molecular docking, potential drugs, bioinformatics

## Abstract

Pulmonary arterial hypertension (PAH) is a devastating cardiovascular disease without a clear mechanism or drugs for treatment. Therefore, it is crucial to reveal the underlying molecular mechanism and identify potential drugs for PAH. In this study, we first integrated three human lung tissue datasets (GSE113439, GSE53408, GSE117261) from GEO. A total of 151 differentially expressed genes (DEGs) were screened, followed by KEGG and GO enrichment analyses and PPI network construction. Five hub genes (CSF3R, NT5E, ANGPT2, FGF7, and CXCL9) were identified by Cytoscape (Cytohubba). GSEA and GSVA were performed for each hub gene to uncover the potential mechanism. Moreover, to repurpose known and therapeutic drugs, the CMap database was retrieved, and nine candidate compounds (lypressin, ruxolitinib, triclabendazole, L-BSO, tiaprofenic acid, AT-9283, QL-X-138, huperzine-a, and L-741742) with a high level of confidence were obtained. Then ruxolitinib was selected to perform molecular docking simulations with ANGPT2, FGF7, NT5E, CSF3R, JAK1, JAK2, JAK3, TYK2. A certain concentration of ruxolitinib could inhibit the proliferation and migration of rat pulmonary artery smooth muscle cells (rPASMCs) *in vitro*. Together, these analyses principally identified CSF3R, NT5E, ANGPT2, FGF7 and CXCL9 as candidate biomarkers of PAH, and ruxolitinib might exert promising therapeutic action for PAH.

## INTRODUCTION

Pulmonary arterial hypertension (PAH) is a serious cardiovascular disease leading to right heart failure and eventually death [1]. The main pathophysiology of PAH is incrassation of the medial and intimal layers of the pulmonary arterial wall, which might result in increased pulmonary vascular resistance and haemodynamic derangements [2]. Although many genes and related biological processes have been reported to be involved in the development of PAH in recent years [3], the underlying molecular mechanism of PAH remains unclear. In terms of PAH therapy, the curative effects of specific drugs related to the prostacyclin pathway [4], endothelin pathway [5] or nitric oxide pathway [6] are not satisfactory following the support of clinical trials consisting of all PAH subtypes. Therefore, investigation of the underlying mechanisms of PAH and a search for auxiliary potential drugs for its treatment are still desperately needed.

Recently, there has been a growing trend for PAH research to utilize high-throughput technologies to explore novel diagnostic or prognostic biomarkers and therapeutic targets. For example, Wang et al. reported that YWHAB was a diagnostic biomarker for idiopathic pulmonary arterial hypertension [3], and Zhu et al. uncovered that miR-140-5p and TNF-α might be therapeutic targets for PAH [7]. Recently, many bioinformatics tools were developed and FocusHeuristics is a competitive approach to explore disease-associated genes [8]. On the other hand, “Cytohubba”, a plug-in of Cytoscape, is a useful and user-friendly tool to obtain hub genes in biological network and has been widely applied [9]. However, the limited sample sizes make these studies inconsistent and can lead to unconvincing conclusions about biological functions. Therefore, it is much more critical to elucidate the underlying molecular mechanism of PAH, which will in turn provide possible routes on which potential treatments can be designed.

Regarding PAH treatment, to develop a novel drug requires substantial costs and a lengthy process of ensuring its safety and tolerance in the human body [10, 11]. By contrast, repurposing a non-novel drug with precise and new mode of action is relatively more cost-efficient and time-saving. Recently, an increasing number of free databases and online tools have been developed, which could help our repositioning of known drugs. For example, the Connectivity map (CMap; https://clue.io/) database serves as a potentially useful tool for drug screening that can predict molecular targeted agents based on DEGs [12]. Combined with high-throughput data, it facilitates the repurposing of known drugs which has passed the toxicology and dosage analyses. Recently, a study reanalysed CMap whole-genome transcriptome data by combining 26 similarity scores with 6 different heuristics. It provided an insight to find a known drug by comparing the effects on the transcriptomes [13]. Then, molecular docking simulation and *in vitro* experiments can be performed to verify the prediction results.

In the present study, we integrated 3 datasets containing 132 human lung tissue samples to obtain DEGs. Then, Kyoto Encyclopedia of Genes and Genomes (KEGG) and Gene Ontology (GO) enrichment analyses were performed based on the DEGs. After obtaining the hub genes from the protein-protein interaction (PPI) network, Gene set enrichment analysis (GSEA) and gene set variation analysis (GSVA) were utilized to further reveal the potential biological functions of the hub genes [14]. Then, the CMap database was employed to search for potential compounds for PAH treatment. Molecular docking simulation between candidate compound and the proteins encoded by hub genes was conducted to validate their prospective application in PAH therapy. Ultimately, *in vitro* experiments verified the effects of ruxolitinib in PAH.

## RESULTS

### Identification of DEGs related to PAH

**Figure 1:**
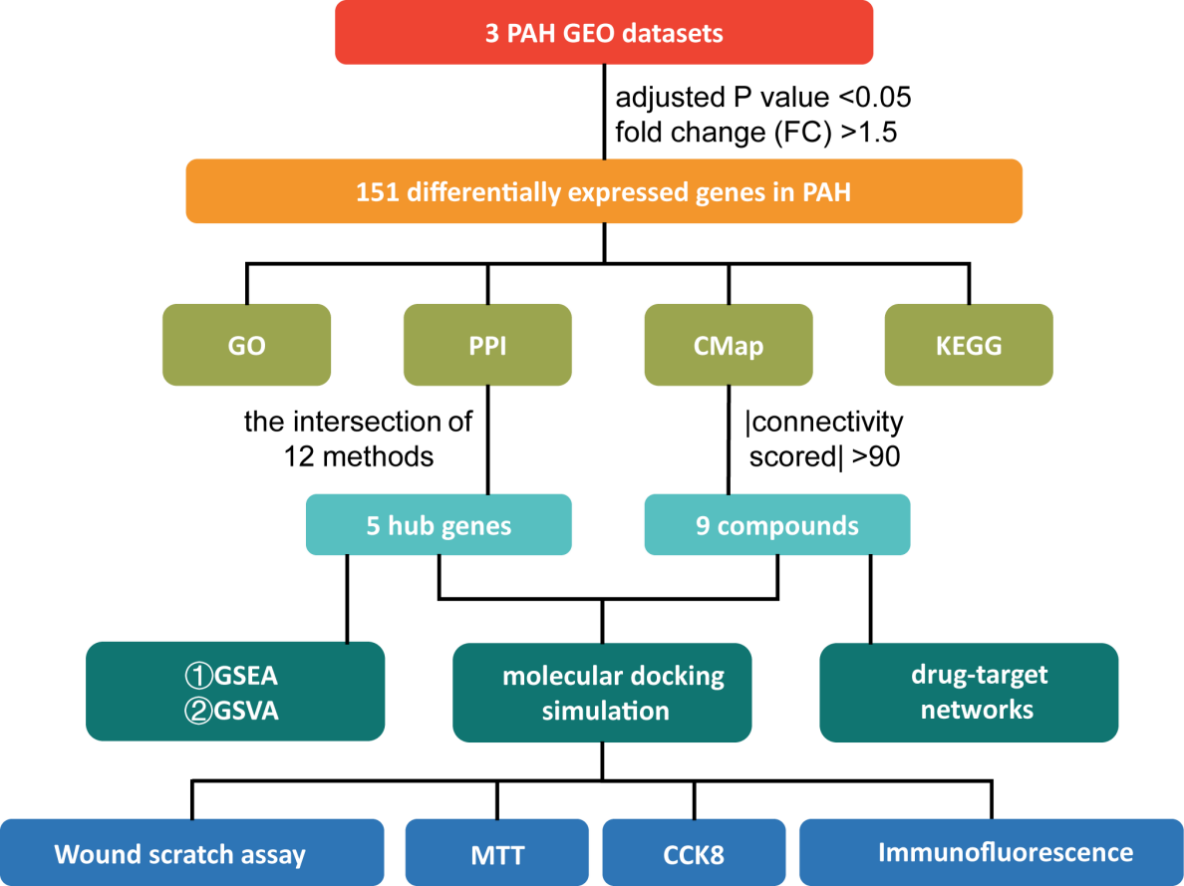
**The study design.** PAH: pulmonary arterial hypertension; GEO: Gene Expression Omnibus; GO: Gene Ontology; PPI: protein-protein interaction; CMap: Connectivity Map; KEGG: Kyoto Encyclopedia of Genes and Genomes; GSEA: Gene Set Enrichment Analysis; GSVA: Gene Set Variation Analysis; MTT: 3-[4,5-Dimethylthiazol-2-yl]-2,5-diphenyltetrazolium Bromide; CCK-8: Cell Counting Kit-8.

**Figure 2:**
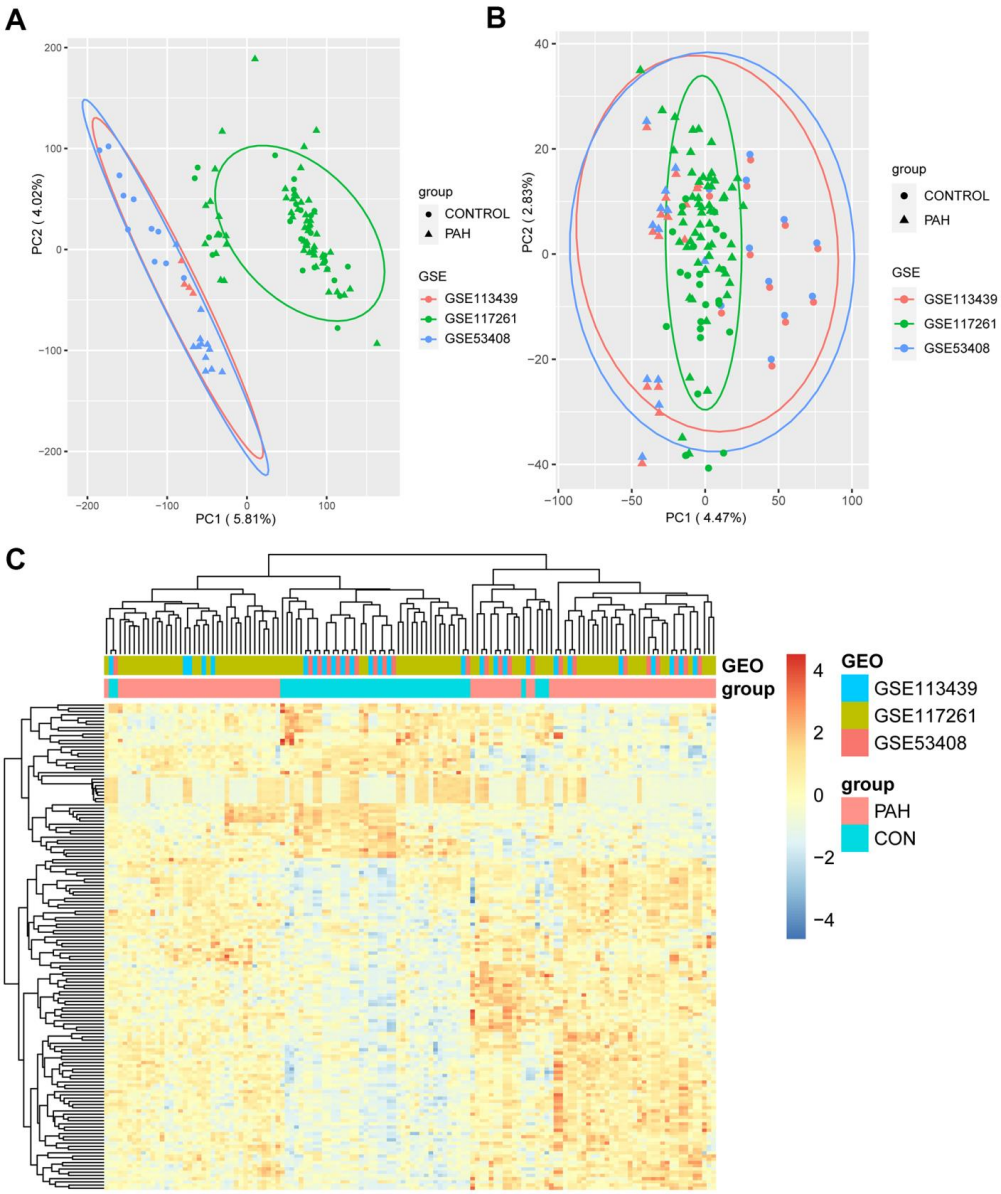
**The result of DEGs identification.** (**A**) Principal component analysis (PCA) before batch effects removement. (**B**) PCA after batch effects removement. (**C**) Heatmap of 151 DEGs screened by “Limma” package. In the differentiating gene sets (GSE113439, GSE117261 and GSE53408), samples were sorted by columns, and genes were sorted by rows. Cyan squares represented the control group, and red squares represented the PAH group. DEGs: differentially expression genes.

The study design is illustrated in Figure 1. We downloaded the raw data of GSE113439, GSE53408, and GSE117261 from the Gene Expression Omnibus (GEO; https://www.ncbi.nlm.nih.gov/gds/) database [15]. Supplementary Table 1 shows the general characteristics of the three datasets. Next, they were inputted into R by “affy” package and the missing data were filled by “impute” package [16]. Then, “sva” package was used to remove batch effects [17]. The Q-Q plot shows the effect of batch removal (Supplementary Figure 1). The heterogeneity of the datasets before and after batch effect removal was examined by “pca” package (Figures 2A, 2B). To annotate the data, we used R software to match each probeset to the corresponding gene symbol according to “hugene10sttranscriptcluster.db” package. If multiple probesets mapped to a same gene symbol, the maximum expression value was selected. In total, 18,820 genes were obtained.

To identify the DEGs, the 132 samples were divided into 47 control samples (CON group) and 85 pulmonary arterial hypertension samples (PAH group). “Limma” package was utilized to conduct the differential gene expression analysis, and a total of 151 significant DEGs (103 upregulated and 48 downregulated) were identified (adjusted P value < 0.05, FC > 1.5; Supplementary Table 2) [18]. All DEGs were used to perform hierarchical clustering analysis, and the heatmap showed evidently different expression between the two groups (Figure 2C).

### GO and KEGG pathway enrichment analyses of DEGs

**Figure 3:**
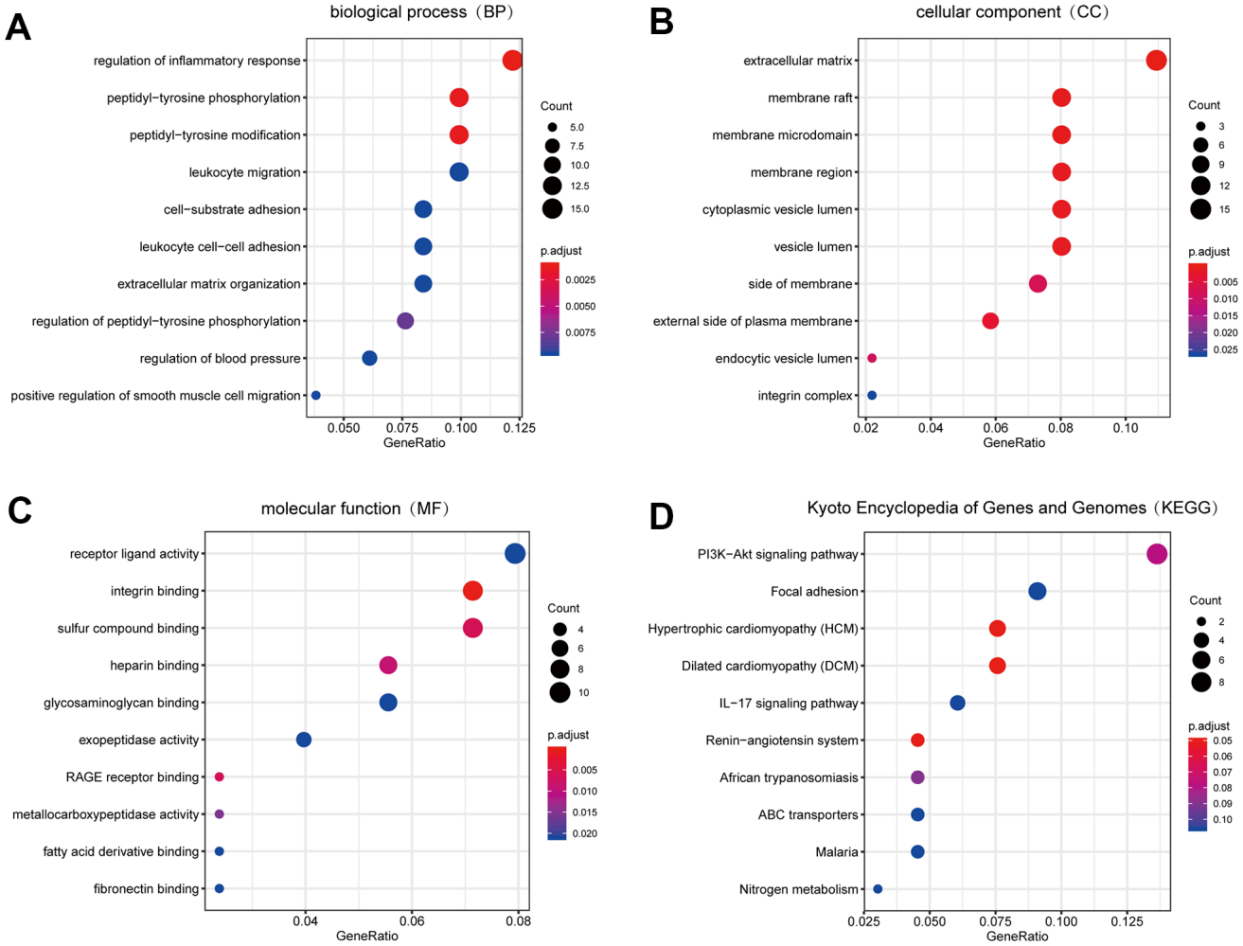
**The result of GO and KEGG pathway enrichment analyses.** (**A**) Biological process GO terms for DEGs. (**B**) Cellular component GO terms for DEGs. (**C**) Molecular function GO terms for DEGs. (**D**) KEGG pathways for DEGs. Top 10 sorted by GeneRatio of GO terms or KEGG pathways were shown. GO: Gene Ontology; KEGG: Kyoto Encyclopedia of Genes and Genomes. DEGs: differentially expression genes.

We carried out GO enrichment analysis on DEGs with the help of “clusterprofiler” package to explore their biological features [19]. Biological process (BP) terms showed that the DEGs were enriched in “regulation of inflammatory response”, “peptidyl-tyrosine phosphorylation” and “peptidyl-tyrosine modification” (Figure 3A), suggesting that the inflammatory response might play an important role in the development of PAH. In terms of cellular component (CC), the terms “extracellular matrix”, “membrane raft” and “membrane microdomain” were significantly enriched (Figure 3B). Therefore, we hypothesized that the DEGs mainly played roles in the extracellular matrix. The major enriched molecular function (MF) terms of the DEGs were “receptor ligand activity”, “integrin binding” and “sulfur compound binding” (Figure 3C).

Moreover, to explore the more profound function of DEGs, we conducted KEGG pathway enrichment analysis with “clusterprofiler” package [19]. Based on the results, the following pathways were significantly enriched in DEGs: “PI3K-AKT signalling pathway”, “focal adhesion” and “hypertrophic cardiomyopathy” (HCM) (Figure 3D).

### PPI network analysis and hub gene recognition

**Figure 4:**
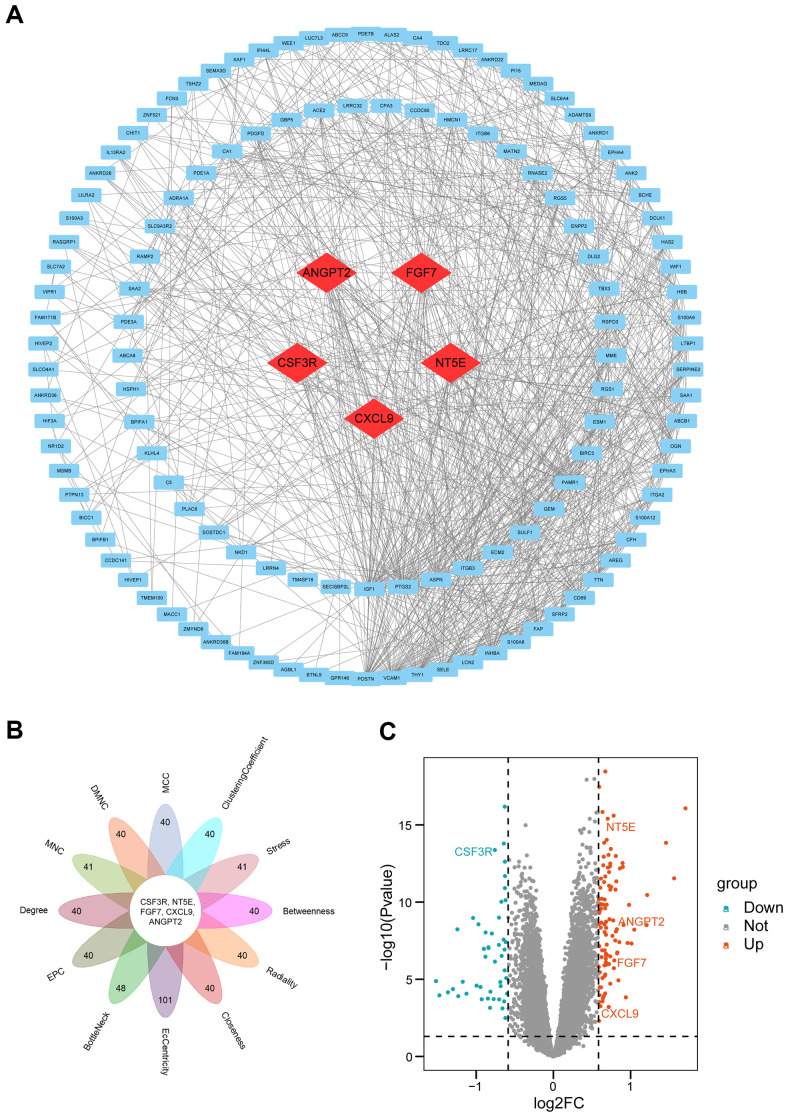
**PPI network construction and hub genes analyses.** (**A**) PPI network. Quadrangles represented proteins and lines represented interactions between proteins. hub genes were in red color. (**B**) Flower plot of results from twelve algorithms. (**C**) Volcano plot of all genes. Orang dots represented 103 up-regulated genes and cyan dots represented 48 down-regulated genes. CSF3R, NT5E, ANGPT2, FGF7, CXCL9 marked in the figure were hub genes. PPI: protein-protein interaction.

To find the hub genes, a PPI network was constructed with Search Tool for the Retrieval of Interacting Genes (STRING; https://string-db.org/) [20], and a total of 128 nodes and 858 edges were in the PPI network (Figure 4A). Next, we took advantage of Cytoscape's plug-in “Cytohubba” to explore the PPI network. To have a more credible result, all 12 algorithms were utilized to calculate the degree of connectivity of DEGs. We selected top 40 genes in each algorithm (As many genes scored equally in the MNC, Stress, BottleNeck, and EcCentricity algorithms, the number of obtained candidate genes was over 40 (Supplementary Table 3)). Then we intersected the results of 12 algorithms. Finally, five hub genes (CSF3R, NT5E, ANGPT2, FGF7, and CXCL9) were obtained (Figure 4B and Supplementary Table 4). The five hub genes are labelled in the volcano plot to show their expression levels (Figure 4C).

### GSEA and GSVA to reveal the potential functions of hub genes

**Figure 5:**
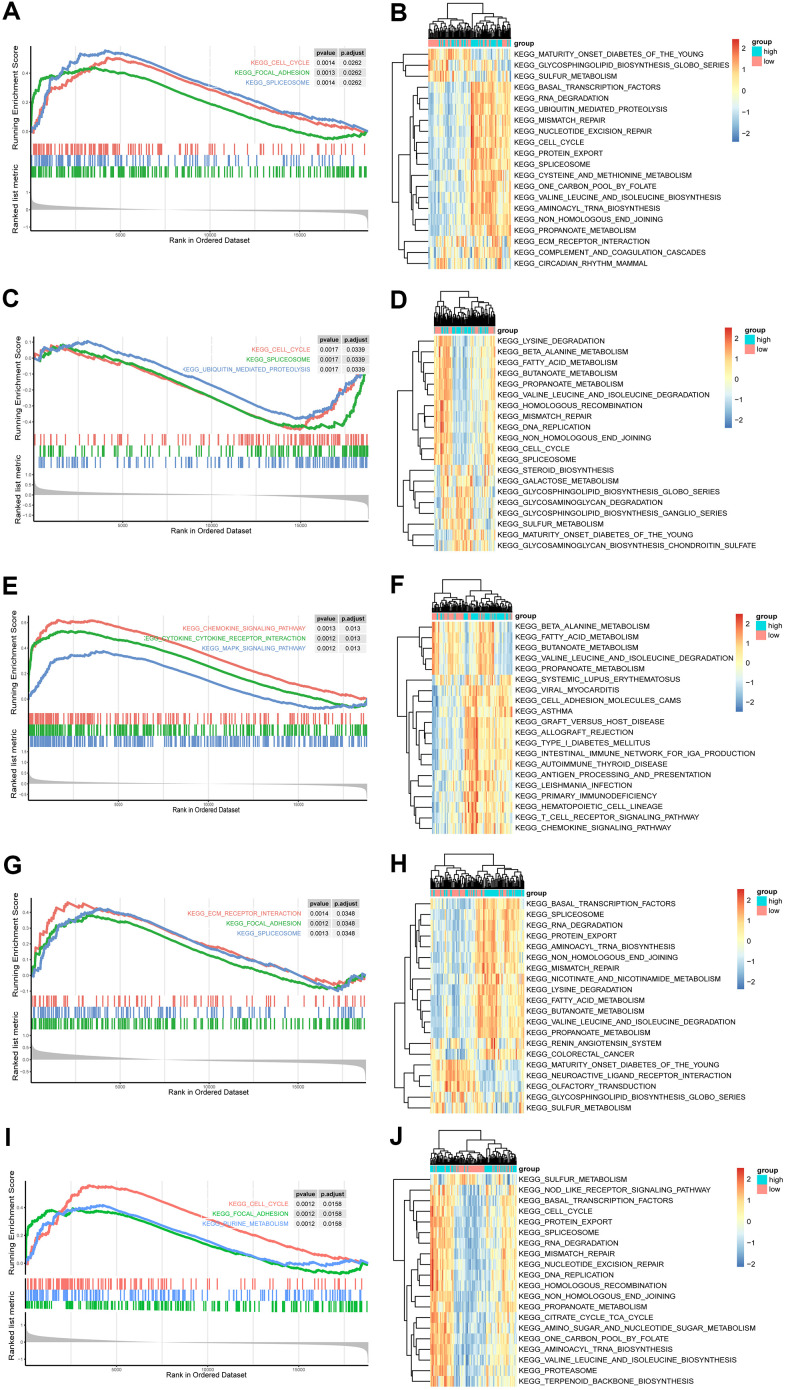
**GSEA and GSVA of hub genes in the PAH.** (**A**, **C**, **E**, **G**, **I**) Top 3 KEGG pathways in the high-expression group of single hub genes. (**A**) ANGPT2; (**C**) CSF3R; (**E**) CXCL9; (**G**) NT5E; (**I**) FGF7. (**B**, **D**, **F**, **H**, **J**) GSVA-derived clustering heatmaps of top 20 differentially expressed pathways for each hub gene. (**B**) ANGPT2; (**D**) CSF3R; (**F**) CXCL9; (**H**) NT5E; (**J**) FGF7.

To further reveal the potential function of the five hub genes, GSEA was performed based on each single hub gene (Figures 5A, 5C, 5E, 5G, 5I). The NT5E, CSF3R and ANGPT2 groups were enriched in the spliceosome pathway. Meanwhile, the cell cycle was enriched in FGF7, ANGPT2 and CSF3R groups, whereas focal adhesion was enriched in the FGF7, ANGPT2 and NT5E groups. The other enriched items were specific to single groups. For example, the chemokine signalling pathway, purine metabolism, cytokine-cytokine receptor interaction and the MAPK signalling pathway were enriched in a group of CXCL9. Most of the pathways were closely associated with cell proliferation.

Furthermore, the top 20 GSVA terms of the five groups are shown in Figures 5B, 5D, 5F, 5H, 5J, and the details of the 20 terms are shown in Supplementary Tables 5–9. The top GSVA term in each result was “one carbon pool by folate”, “propanoate metabolism”, “graft versus host disease”, “valine leucine and isoleucine degradation”, and “protein export”. In summary, these results confirmed that these hub genes contributed to proliferation processes.

### CMap analysis and molecular docking simulation

To find drugs for PAH therapy, we searched the CMap database. Nine candidate compounds (lypressin, ruxolitinib, triclabendazole, L-BSO, tiaprofenic acid, AT-9283, QL-X-138, huperzine-a, and L-741742) were considered with high levels of confidence (Table 1). Their 2D structures were provided by PubChem (https://pubchem.ncbi.nlm.nih.gov/; Supplementary Figure 2) [21]. Because the 2D structure of QL-X-138 was not retrieved, only eight 2D structures are presented. The targets of these nine compounds provided by the CMap database were utilized to construct drug-target networks in Cytoscape software (Supplementary Figure 3).

**Table 1:** The result of CMap.

**Drug name**	**Score**	**Description**	**Target**
lypressin	98.84	Vasopressin receptor agonist	AVPR1A, AVPR1B, AVPR2
ruxolitinib	97.86	JAK inhibitor	JAK1, JAK2, TYK2, JAK3
triclabendazole	94.73	Microtubule inhibitor	DNMT1
L-BSO	94.66	Glutathione transferase inhibitor	GCLM
tiaprofenic-acid	93.96	Cyclooxygenase inhibitor	PTGS2, PTGS1
AT-9283	91.00	JAK inhibitor	AURKA, AURKB, ABL1, BCR, FLT3, JAK2, JAK3, RPS6KA6, STK17A
QL-X-138	90.35	MTOR inhibitor	BTK, JAK3, MKNK2, MTOR, PRKDC
L-741742	-90.13	Dopamine receptor antagonist	DRD4, DRD3, SCN1A, SCN3A
huperzine-a	-90.46	Acetylcholinesterase inhibitor	ACHE

**Figure 6:**
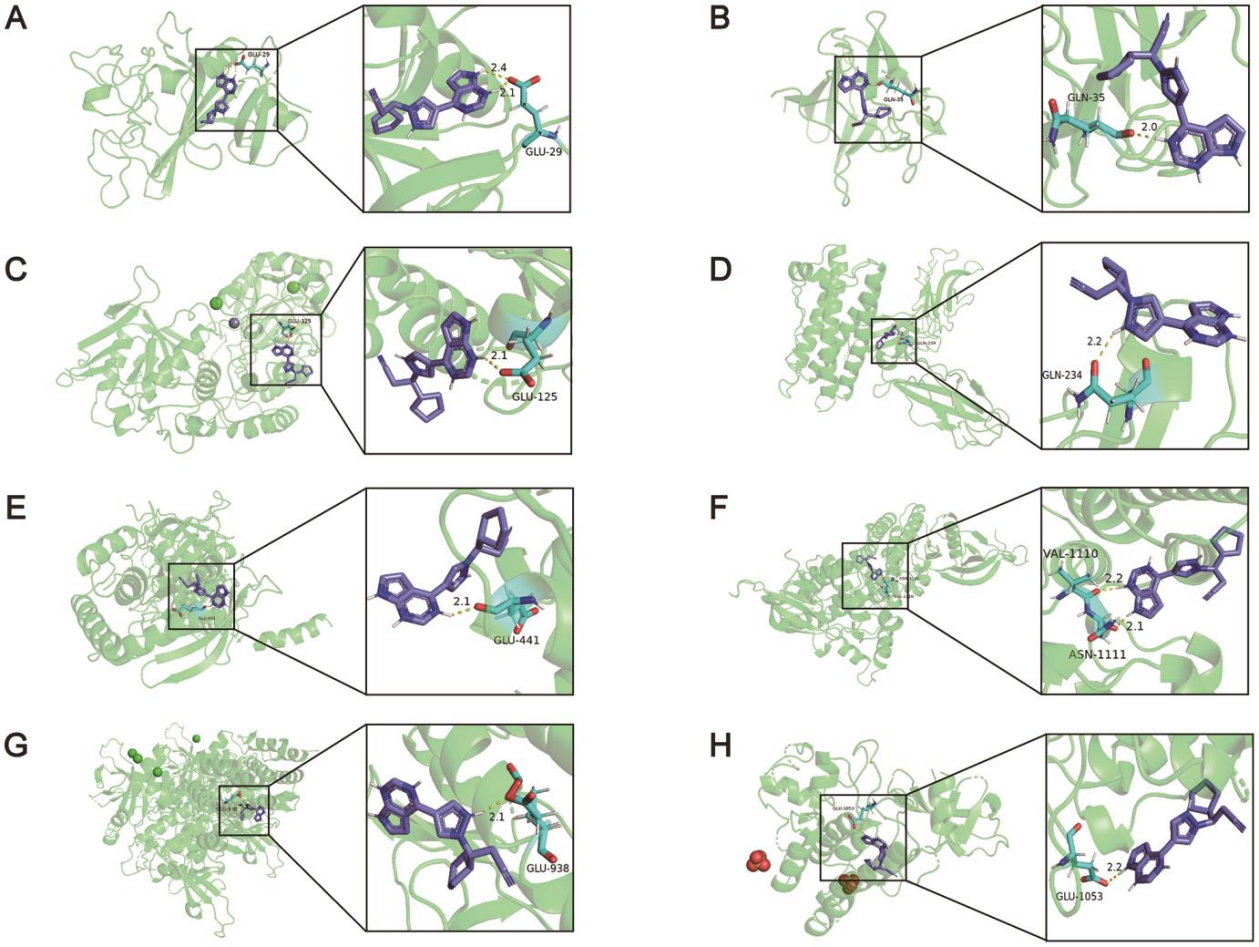
**The results of the molecular docking simulations.** (**A**) There were two hydrogen bonding between the amino acid residue of ANGPT2 (PDB: 4JZC) (GLU29) and ruxolitinib, and the distance between the atoms was 2.1Å and 2.4Å. (**B**) The amino acid residue of FGF7 (PDB: 1QQL) bound to ruxolitinib was GLN35, and the distance was 2.0Å. (**C**) The amino acid residue of NT5E (PDB: 4H2F) bound to ruxolitinib was GLU125, and the distance was 2.1Å. (**D**) The amino acid residue of CSF3R (PDB: 2D9Q) bound to ruxolitinib was GLN234, and the distance was 2.2Å. (**E**) The amino acid residue of JAK1 (PDB: 4GS0) bound to ruxolitinib was GLU441, and the distance was 2.1Å. (**F**) The amino acid residues of JAK2 (PDB: 2B7A) bound to ruxolitinib were VAL1110 and ASN1111, and the distance were 2.2Å and 2.1Å. (**G**) The amino acid residue of JAK3 (PDB: 3ZC6) bound to ruxolitinib was GLU938, and the distance was 2.1Å. (**H**) The amino acid residue of TYK2 (PDB: 4GFO) bound to ruxolitinib was GLU1053, and the distance was 2.2Å.

Furthermore, molecular docking simulation was utilized to delve into the possible therapeutic mechanisms of these drugs. Five hub genes were supposed to serve as potential therapeutic targets in PAH. Among the nine compounds, lypressin ranked first, but its 3D structure was not provided on ZINC (http://zinc.docking.org/) [22]. In addition, ruxolitinib, described as a Janus-associated kinase (JAK) inhibitor in the CMap database, obtained the second highest score (97.86). Activation of the JAK-STAT signalling pathway has been reported to induce the transcription of pro-angiogenesis and pro-inflammatory genes, leading to the progression of PAH [23]. Consequently, ruxolitinib was selected to dock with the proteins encoded by hub genes. We downloaded the 3D structures of NT5E (PDB: 4H2F), ANGPT2 (PDB: 4JZC), FGF7 (PDB: 1QQL), CSF3R (PDB: 2D9Q) and ruxolitinib to perform the molecular docking simulations, whereas the 3D structure of CXCL9 was not provided on RCSB PDB (https://www.rcsb.org/) [24]. In addition, JAK1 (PDB: 4GS0), JAK2 (PDB: 2B7A), JAK3 (PDB: 3ZC6) and TYK2 (PDB: 4GFO) as known targets of ruxolitinib served as positive controls. The binding energies between ruxolitinib and ANGPT2, FGF7, NT5E, CSF3R, JAK1, JAK2, JAK3, TYK2 were -6.42, -5.25, -6.30, -3.69, -3.59, -3.65, -3.90, -4.73 kcal/mol, respectively (Figure 6). It indicated that ANGPT2, FGF7 and NT5E may be the targets of ruxolitinib in treatment of PAH (The binding energies of these 3 proteins were higher than the positive controls). Other details of the results, such as the hydrogen bond, atomic distance and binding site data, are shown in Figure 6. From the above findings, ruxolitinib may have an unexpected effect in the treatment of PAH.

### Ruxolitinib significantly inhibits the proliferation and migration abilities of hypoxia-induced rPASMCs

**Figure 7:**
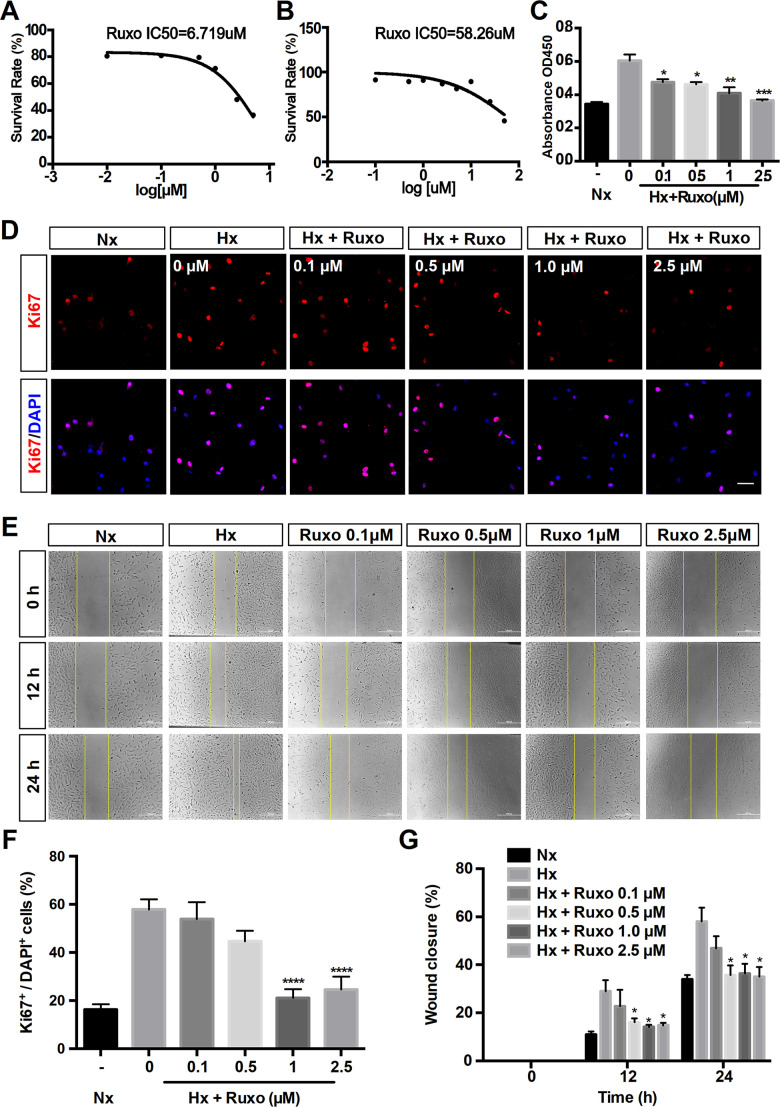
**Ruxolitinib inhibits hypoxia-induced rPASMCs proliferation and migration.** Nx = Normoxia group; Hx = Hypoxia group; Hx + Ruxo = Hypoxia plus Ruxolitinib group. (**A**, **B**) Determination of IC50 values. RPASMCs were incubated with ruxolitinib at different concentrations (0, 0.1, 0.5, 1 and 2.5 μM) for 24 h under hypoxia (**A**) and normoxia (**B**), viable cell number was determined by MTT assay. The results yielded IC50 values were 6.719 μM (**A**) and 58.26 μM (**B**), respectively. (**C**) CCK-8 assay for cell proliferation. RPASMCs were treated with indicated concentrations of ruxolitinib for 24 h, and the cell proliferation was determined by CCK-8 assay. (**D**, **F**) Immunofluorescence staining of Ki67(red) in rPASMCs of indicated groups. RPASMCs were treated with or without indicated concentrations of ruxolitinib for 24 h under hypoxic conditions, the untreated cells were treated as a normoxia control group. Cells nuclear were counterstained with DAPI (blue). (**E**, **G**) Wound scratch assay. RPASMCs were treated with ruxolitinib at a specified concentration in the presence or absence of oxygen for 12h and 24h, migration capabilities were represented by relative migration distances. Data are presented as mean ± SEM, *P < 0.05, **P < 0.01, ***P < 0.001, ****P < 0.0001.

To further explore the effects of ruxolitinib in PAH, we investigated the regulation of rPASMCs migration and proliferation by ruxolitinib. Firstly, the cytotoxicity of ruxolitinib was assessed by 3-[4,5-Dimethylthiazol-2-yl]-2,5-diphenyltetrazolium Bromide (MTT) assay and evaluated by the 50% inhibitory concentration (IC50) values (6.719 μM under hypoxic and 58.26 μM under normoxic) (Figures 7A, 7B). Therefore, the concentrations of ruxolitinib was used including 0, 0.1, 0.5, 1 and 2.5 μM. The result of CCK-8 assay indicated the inhibited proliferation in PASMCs with the treatment of ruxolitinib (Figure 7C). With concentration increasing, the inhibition effect became more obvious. Coincidentally, immunofluorescence assay demonstrated that administration of ruxolitinib significantly decreased the expression of Ki67 (a marker of proliferation) in hypoxia-induced rPASMCs (Figures 7D, 7F). Moreover, scratch wound assay showed that rate of closure was increased under hypoxia exposure for 12h and 24h, nevertheless hypoxia-induced migration was significantly suppressed by ruxolitinib (Figures 7E, 7G). Collectively, these data strongly suggest that ruxolitinib could effectively inhibit cell proliferation and cell migration under hypoxia exposure *in vitro*.

## DISCUSSION

Here, we identified five hub genes of PAH and potential mechanism of PAH with bioinformatic analyses. Further studies are urgently needed to verify the hub genes. Furthermore, nine candidate compounds were predicted through the CMap database, in which ruxolitinib was considered the drug with the most clinical potential given its ability to target ANGPT2, FGF7 and NT5E, especially ANGPT2 and inhibit proliferation and migration of hypoxia-induced rPASMCs. These findings will shed new light on promising therapeutic strategies to treat PAH.

PAH is a serious pulmonary vascular disease caused by multiple risk factors [17]. Although great progress has been made in the study of PAH, there is still a lack of effective methods to diagnose and treat PAH. Fortunately, with the development of high-throughput technologies, an increasing number of novel biomarkers and therapeutic targets for PAH have been uncovered. For example, Sun et al. found that Smad9, BMPR2, Eng, and IL4 were differentially expressed in PAH mice. On the other hand, lncRNAs NR-036693, NR-027783, NR-033766, and NR-001284 played an important role in PAH pathology and might serve as therapeutic targets for PAH. In the present study, we integrated three human lung tissue datasets, obtaining 151 significant DEGs to explore the critical genes, pathways and potential drugs that are associated with PAH.

According to the integrated expression data, 151 DEGs were identified between the CON group and PAH group. The results of the BP term GO annotation analysis indicated that the DEGs were markedly enriched in the inflammatory response, which was consistent with the previous demonstration that inflammation was emerging as a key disease-related factor in PAH [25]. The enriched CC terms of DEGs were related to the extracellular matrix, which was in accordance with published literature showing that the extracellular matrix plays a central role in the pathogenesis of PAH [26]. The MF analysis of GO terms revealed that the DEGs were the most significantly enriched in receptor ligand activity, suggesting that receptor-ligand interactions are an essential component of PAH [27]. Additionally, KEGG analysis suggested that the DEGs were the most significantly enriched in the PI3K-AKT signalling pathway, which was consistent with the previous demonstration that pulmonary hypertension involves crosstalk with proliferation and apoptosis mechanisms [28, 29]. The results of the enrichment analysis exactly support the validity of the DEGs obtained in the previous analysis.

In the present study, five hub genes (FGF7, CXCL9, NT5E, ANGPT2 and CSF3R) were identified. Among them, FGF7 was upregulated in PAH and confirmed to be inhibited by miR-455-3p-1 via the RAS/ERK signalling pathway [30]. Belperio et al. showed that there was no significant difference in CXCL9 expression in the plasma of normal and PAH patients [31]. However, in this study, we used a much larger sample size and selected human lung tissue samples, which confirmed that CXCL9 was expressed at higher levels in the lung tissue of PAH patients than in the lung tissues of healthy controls. Moreover, ANGPT2 was upregulated in chronic obstructive pulmonary disease (COPD) patients with PAH [32]. However, the specific role of ANGPT2 in the progression of PAH is still unclear. As far as the other hub genes, the roles of NT5E and CSF3R have not been identified. Therefore, exploring the functions of these genes could provide useful information.

To further explore the role of the hub genes in the progression of PAH, GSEA and GSVA were performed based on each hub gene. GSEA is a powerful analytical method using the cross-exchange hypothesis test model to evaluate the score of the gene sets and can reveal many related biological pathways [14]. In addition, GSVA is another gene set enrichment analysis method that can estimate the variation of pathway activity over every sample in an unsupervised manner and further identify related gene sets [33]. By combining the two enrichment analytical methods, we were able to obtain more credible results. In the present research, many cell cycle-related pathways were enriched, including cell cycle, DNA replication and mismatch repair pathways, suggesting that the genes involved in these pathways may contribute to proliferation processes.

In the present study, we found nine potential small molecular compounds that can reverse the altered expression of the DEGs and improve PAH through CMap analysis. Among the nine compounds, lypressin is an antidiuretic hormone that has been found in pigs and some marsupial families [34]. Triclabendazole is an antihelminthic drug and is used in the treatment of fascioliasis [35]. Tiaprofenic acid is a non-steroidal anti-inflammatory drug that is widely used to treat pain, particularly arthritis pain [36]. As a multitarget kinase inhibitor, AT-9283 can inhibit the process of STAT3 tyrosine phosphorylation and inhibit multiple myeloma cell proliferation [37] and is widely used in cancer treatment [38, 39]. Another study showed that AT-9283 is an aurora kinase/JAK inhibitor [40]. Huperzine-a is reported as acetylcholinesterase inhibitor and neuroprotective agent [41, 42]. L-741742 is a dopamine receptor antagonist [43]. Nevertheless, previous reports have not shown whether these drugs are effective in treating PAH.

Overall, ruxolitinib obtained the second highest score (97.86) and targets JAK1, JAK2, TYK2 and JAK3 based on the CMap database. Previously, ruxolitinib was confirmed to be used to treat myelofibrosis [44]. Epidemiological studies found PAH to be a common complication of myelofibrosis [45], and ruxolitinib could effectively improve PAH in myelofibrosis patients [46]. However, a case report reported that ruxolitinib might exacerbate PAH, but its mechanism was unclear [47]. To further explore the interaction between ruxolitinib and PAH, we performed molecular docking simulations between ruxolitinib and the proteins encoded by hub genes (ANGPT2, FGF7, NT5E, and CSF3R). Among the hub genes, CXCL9 was not considered due to the lack of 3D structure. The results showed that ruxolitinib has good binding ability with ANGPT2, FGF7 and NT5E, suggesting that ruxolitinib has potential utility in PAH therapy. Therefore, *in vitro* experiments were conducted. The obtained results displayed the significantly suppressive effect of ruxolitinib on rPASMCs proliferation and migration. Interestingly, as an inhibitor of JAK, ruxolitinib significantly reduced cytokine-mediated lung adenocarcinoma proliferation by inhibiting the JAK/STAT signal pathway [48]. Similarly, in the basic study of JAK2-V617F-positive leukemia cells, ruxolitinib may inhibit cell proliferation through dephosphorylation of the JAK2 substrate STAT5 and further regulation of the mTORC1/S6K/4EBP1 signal pathway [49]. This study was highly correlated with our results of enrichment analysis (PI3K-AKT signalling pathway). Recently, a study reported ruxolitinib has a therapeutic effect on PAH through blocking Jak2-Stat3 signalling pathway [50]. The view in this article supports our result that ruxolitinib might exert promising therapeutic action for PAH. However, relevant molecular mechanisms that the article has studied are different than the ones we explored. In present study, the proteins encoded by 3 hub genes (ANGPT2, FGF7, NT5E) were considered as the targets. Meanwhile, ruxolitinib acted by suppressing the proliferation via PI3K-AKT signalling pathway. These demonstrated that ruxolitinib may have particular value in the treatment of PAH. However, the effects and mechanisms of ruxolitinib should still be validated by further experimental evidence and long-term clinical trials.

## MATERIALS AND METHODS

### Identification of DEGs

The raw data of three eligible microarray datasets (GSE113439, GSE53408, GSE117261) based on platform GPL6244 (Affymetrix Human Gene 1.0 ST Array) were downloaded from GEO database. Background correction, normalization and expression calculation of the raw data were carried out with “affy” package using the Robust Multi-array Average (RMA) method [51]. Then the “Knn” method in “impute” package was used to fill the missing data. To remove the batch effects, “sva” package in R software was applied. We matched the probesets with corresponding gene symbols with “hugene10sttranscriptcluster.db” package in R software. To select the DEGs, “Limma” package was utilized with cut-offs of adjusted P value < 0.05 and fold change (FC) > 1.5.

### GO and KEGG pathway enrichment analyses

GO enrichment analysis is a commonly used bioinformatic approach for searching comprehensive information of large-scale gene data, including BP, CC and MF. KEGG pathway enrichment analysis is widely used to understand biological mechanisms and functions. GO and KEGG enrichment analyses were performed with “clusterprofiler” package. The top 10 genes sorted by the GeneRatio of GO terms and KEGG pathways were visualized by “GOplot” package [47].

### PPI network construction and hub gene identification

A PPI network of DEGs was constructed by the STRING database with a combined score > 0.7 as the cut-off point to assess the direct and indirect associations of the DEGs. “Cytohubba” (a plug-in) was utilized to identify the hub genes in Cytoscape (version 3.7.1). Then, 12 algorithms (MCC, DMNC, MNC, Degree, EPC, BottleNeck, Eccentricity, Closeness, Radiality, Betweenness, Stress and Clustering coefficient) in “Cytohubba” were used to calculate the weight of each gene in total.

### GSEA and GSVA

The “clusterprofile” package was utilized to perform GSEA of the hub genes with integrated gene expression data. PAH samples were divided into two groups (high expression and low expression) based on the median expression of each hub gene. Then differential expression analysis was performed with “Limma” package. The log2 fold change is used as the rank list for GSEA analysis [52]. Finally, the top 3 GSEA terms was visualized by “enrichplot” package. Additionally, “GSVA” package was used to further explore the significant signalling pathways associated with the hub genes [33]. GSVA requires two files, a gene set and a gene expression matrix. “GSVAdata” package was used to read a file of gene set in GMT format. Then, the gene set and the gene expression matrix were integrated by “GSVA” package. Next, a matrix of KEGG pathway was obtained. Furthermore, “Limma” was used to find significant KEGG pathway. “C2.cp.kegg.v7.0.symbols.gmt” served as the reference gene set for both GSEA and GSVA, which was downloaded from MSigDB (http://software.broadinstitute.org/gsea/msigdb/index.jsp) [53]. The top 20 GSVA terms were visualized by “pheatmap” package. P value < 0.05 was considered statistically significant.

### Prediction of potential drugs for PAH treatment

The CMap database is an online resource that can be used to establish links between genes, compounds and diseases based on similar and opposite gene expression profiles. In the present study, the DEGs of PAH were divided into two groups (upregulated genes and downregulated genes). Then, the DEGs were loaded into the “QUERY” page. In this study, connectivity scores > 90 or < -90 were selected. 2D structures of candidate compounds were obtained from PubChem. To further investigate the specific mechanism of the compounds, the molecular docking simulation was performed on AutoDock 4 software (version 4.2.6), which was designed to forecast how small molecules bind to a receptor of known 3D structure [54]. The 3D structures of proteins and compounds were downloaded from RCSB PDB and ZINC, respectively. Then, PyMOL was utilized to remove the water molecules and separate proteins from small molecules. Next, we used AutoDock to prepare the specific coordinate file (PDBQT). Then, the file was implemented to run AutoGrid and AutoDock. We selected the molecular docking of minimal binding energy with hydrogen bonds and exported the results as a PDBQT file. The file was converted into a PDB file by OpenBabel software (version 2.4.1). Finally, PyMOL software (version 2.3) was used to view and visualize the results [55].

### Chemical reagents

Ruxolitinib was purchased from Selleck (Selleck, Houston, TX, USA). MTT powder was purchased from Solarbio company (Solarbio Co., Beijing, China), dissolved by ultrasonic instrument, and finally prepared into MTT reagent (50mg/ mL, PBS). DMSO, DMEM, fetal bovine serum (FBS) were obtained from Thermo Fisher (Thermo Scientific Fisher, Wilmington, USA).

### Extraction and culture of rPASMCs

Sprague-Dawley rats were anesthetized, immobilized, shaved, and disinfected. After opening the thorax of the rats, the heart and lungs were excised and the pulmonary artery was extracted. The pulmonary artery was transferred to high-pressure treated PBS for cleaning and the membrane and intima were removed carefully [56]. The pulmonary medium membrane was cut into small pieces and then cultured in DMEM containing 10% FBS (5% CO_2_ in 37° C). The primary rPASMCs were passaged after being cultured for 1 week. When the rPASMCs confluence reached 80% ~ 90%, they were subcultured to new petri dishes using trypsin–EDTA. The third to the fifth passage of rPASMCs were selected for the following experiments.

### MTT assay

To explore the inhibition effect of ruxolitinib on hypoxic rPASMCs, we utilized MTT cytotoxicity assays to perform the cell viability tests [57]. RPASMCs were inoculated into 96-well plates with 8000-10000 cells/well and allowed to attach for 6-8 h. RPASMCs were treated with ruxolitinib of different concentrations (from 5 mM to 0.1 nM) under hypoxia conditions to obtain the IC50 values. After 24 h of modelling, MTT reagent (5 mg/ml, 25 μl) was added into each well and incubated for another 4 h. We discarded all the medium from the wells, next, added DMSO (150 μl) into each well to dissolve formazan crystals. Then the absorbance at 490 nm was detected by a microplate reader and the IC50 value was calculated by linear regression analysis.

### Examination of cell proliferative ability

Cell proliferation assay was measured by Cell Counting Kit-8 (CCK-8) assay (Dojindo, Kumamoto, Japan). We seeded the rPASMCs into 96-well plates (8000-10000 cells/well) and preincubated until the cells attached. Then, rPASMCs were treated with different concentrations of ruxolitinib (0, 0.1, 0.5, 1 and 2.5 mM) and incubated for 24 h (5% O_2_, 5% CO_2_ in 37° C). Whereafter, we added 10 ul of CCK-8 assay solution to each well and the incubation continued for 4 h. The capacity of cell proliferation was determined by the absorbance measured at 450 nm through a microplate reader.

### Examination of cell migratory ability

Wound scratch assay was performed to determine the ability of cell migratory. RPASMCs were plated onto 12-well plates at a density of 6~8×10^3^ cells/well and cultured up to 90% cell density. Then the streak wounds were scratched with a sterile 20 μl pipette tip. The cell culture medium was changed and treated with different concentrations of ruxolitinib (0, 0.1, 0.5, 1 and 2.5 mM). We photographed the wounds in the same view at 0, 12 and 24 h time points. The relative distance of cell migration was exhibited and measured by ImageJ software (National Institutes of Health, Bethesda, MD).

### Immunofluorescence

To measure the effect of ruxolitinib on the cellular proliferation phenotype, we performed cell immunofluorescence analyses to detect the expression of Ki67 in rPASMCs. RPASMCs were planted on a glass cover slip, fixed with 4% paraformaldehyde and washed with PBS. Respectively, we used 0.1% Triton X-100 and 5% BSA to permeabilize cell membranes and to block cells at room temperature. After the blocking solution was washed off, Ki67 (1:500, AF0198; Affinity) antibodies were used as primary antibody and slides were incubated overnight at 4° C. The next day, cells were incubated with the donkey anti-rabbit (1:1000; Alexa Fluor 594) for 1 h after being washed 3 times. Nuclei were counterstained with DAPI stain for 5 min. Images were observed with a fluorescence microscope (Olympus, Tokyo, Japan).

### Statistical analysis

Statistical analyses were performed with GraphPad Prism 6.0 (GraphPad Software, CA, USA). All the results presented were represented from at least 3 independent experiments. All the data were expressed as the mean standard error of mean (SEM). Comparisons between two groups were analysed by unpaired two-tailed Student’s t-test, and multiple comparisons were analysed by one-way analysis of variance (ANOVA). P values of <0.05 were considered statistically significant.

## Supplementary Material

Supplementary Figures

Supplementary Table 1

Supplementary Table 2

Supplementary Tables 3 to 9
