# Supplementary material for: Integrated bioinformatic analysis reveals the underlying molecular mechanism of and potential drugs for pulmonary arterial hypertension
Source: Aging (Albany NY). 2021 May 18;13(10):14234–57. doi: 10.18632/aging.203040 (PMC8202883; doi:10.18632/aging.203040)
Supplement: Supplementary Figures [file aging-13-203040-s001.pdf]

## SUPPLEMENTARY FIGURES

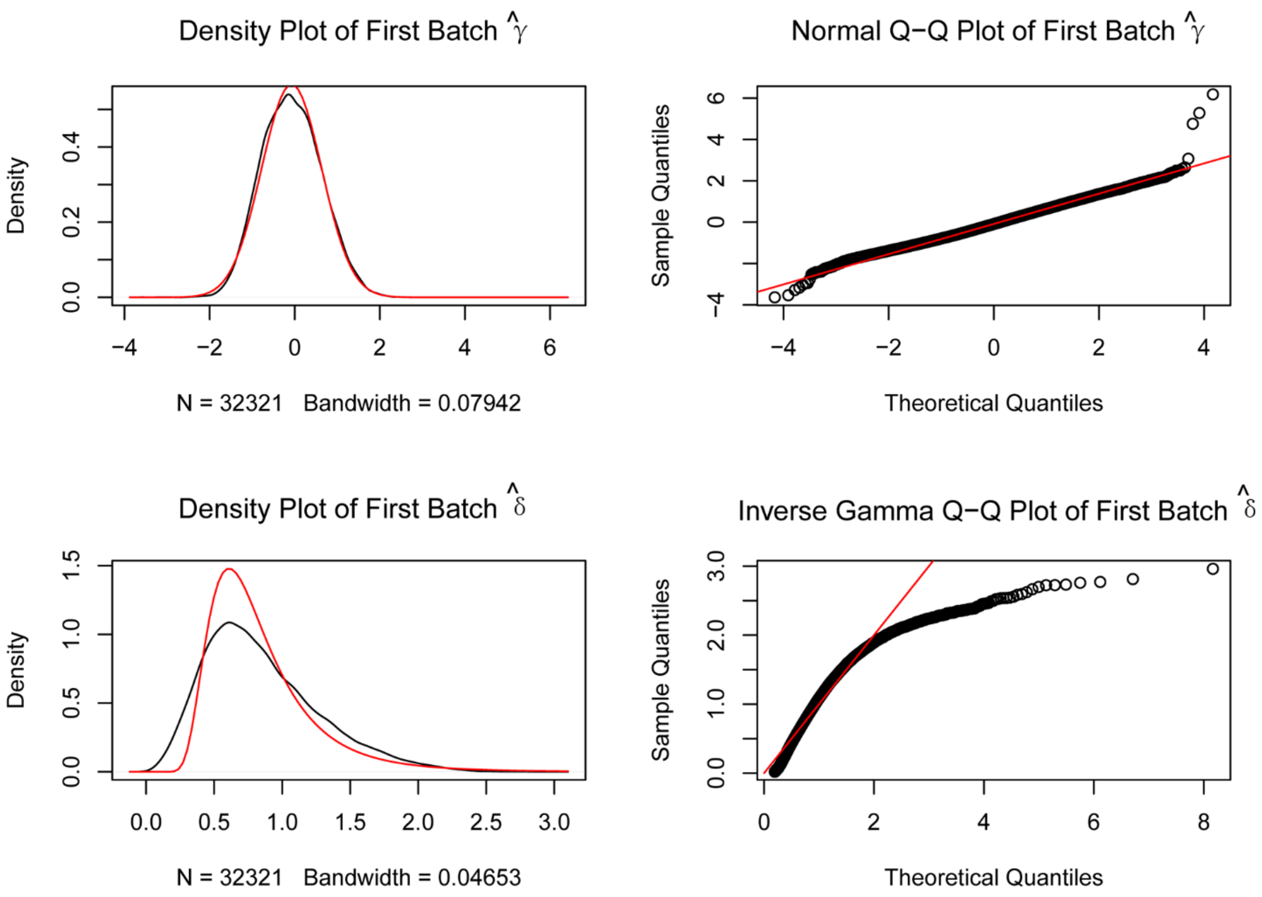


**Supplementary Figure 1. The quantile-quantile (Q-Q) plot of the batch effect of GSE113439, GSE53408 and GSE117261 datasets is removed.**

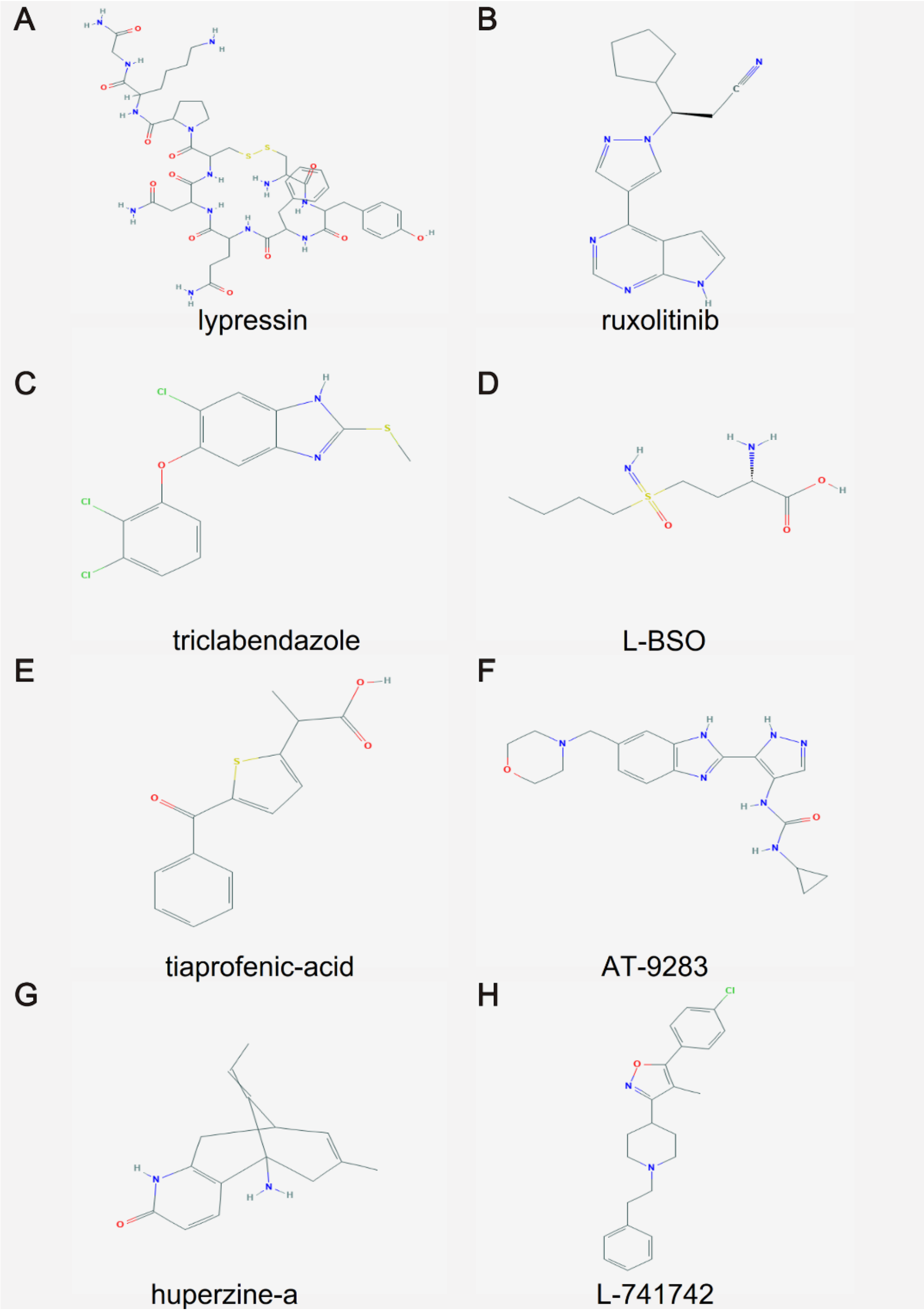


**Supplementary Figure 2. 2D structure of the candidate compounds.** (**A**) lypressin, (**B**) ruxolitinib, (**C**) triclabendazole, (**D**) L-BSO, (**E**) tiaprofenic acid, (**F**) AT-9283, (**G**) huperzine-a, (**H**) L-741742.

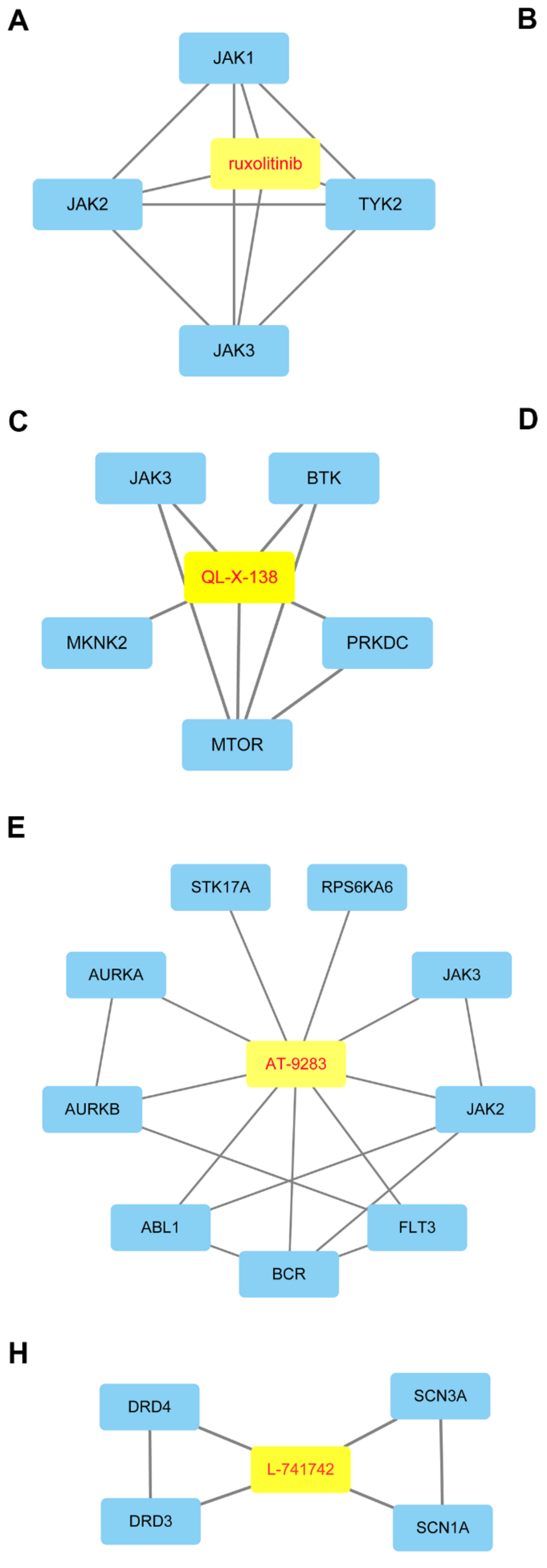


**Supplementary Figure 3. The drug-target network for nine candidate drugs.** Yellow represented drug and blue represented target. (**A**) ruxolitinib. (**B**) lypressin. (**C**) QL-X-138. (**D**) tiaprofenic-acid. (**E**) AT-9283. (**F**) triclabendazole. (**G**) L-BSO. (**H**) huperzine-a. (**I**) L-741742.
