# Supplementary material for: Integrated bioinformatic analysis reveals the underlying molecular mechanism of and potential drugs for pulmonary arterial hypertension
Source: Aging (Albany NY). 2021 May 18;13(10):14234–57. doi: 10.18632/aging.203040 (PMC8202883; doi:10.18632/aging.203040)
Supplement: Supplementary Table 1 [file aging-13-203040-s002.pdf]

SUPPLEMENTARY TABLE

Supplementary Table 1. Characteristics of the individual dataset.

GEO ID	Platform	Tissue type	Sample size	Country	Time
GSE113439	GPL6244 (Affymetrix Human Gene 1.0 ST Array)	human lung tissue	15 vs. 11	Canada	2018
GSE53408	GPL6244 (Affymetrix Human Gene 1.0 ST Array)	human lung tissue	12 vs. 11	Canada	2013
GSE117261	GPL6244 (Affymetrix Human Gene 1.0 ST Array)	human lung tissue	58 vs. 25	United States	2018
