# Supplementary material for: Integrated bioinformatic analysis reveals the underlying molecular mechanism of and potential drugs for pulmonary arterial hypertension
Source: Aging (Albany NY). 2021 May 18;13(10):14234–57. doi: 10.18632/aging.203040 (PMC8202883; doi:10.18632/aging.203040)
Supplement: Supplementary Table 2 [file aging-13-203040-s003.docx]

**Supplementary Table 2. All differentially expressed genes.**

|  | **logFC** | **AveExpr** | **t** | **P.Value** | **adj.P.Val** | **B** |
| --- | --- | --- | --- | --- | --- | --- |
| POSTN | 1.710847 | 9.306826 | 9.542733 | 8.44E-17 | 2.65E-13 | 27.60976 |
| PI15 | 1.563826 | 6.202067 | 7.683117 | 2.88E-12 | 7.32E-10 | 17.51753 |
| RPS4Y1 | -1.51999 | 6.509525 | -4.52544 | 1.31E-05 | 0.000121 | 2.812716 |
| DDX3Y | -1.47618 | 6.214055 | -3.9863 | 0.00011 | 0.000657 | 0.817614 |
| HBB | 1.458893 | 10.41608 | 8.640389 | 1.44E-14 | 1.23E-11 | 22.63899 |
| UTY | -1.36542 | 5.794362 | -4.10395 | 7.00E-05 | 0.00046 | 1.236467 |
| BPIFB1 | -1.30034 | 7.817914 | -4.23109 | 4.27E-05 | 0.000311 | 1.699684 |
| SOSTDC1 | -1.24362 | 7.062101 | -6.22067 | 5.83E-09 | 2.84E-07 | 10.17861 |
| EIF1AY | -1.23169 | 5.126323 | -3.95243 | 0.000124 | 0.00073 | 0.698786 |
| VCAM1 | 1.214823 | 7.476238 | 7.219419 | 3.46E-11 | 5.17E-09 | 15.11585 |
| SFRP2 | 1.207478 | 8.217039 | 6.347798 | 3.10E-09 | 1.66E-07 | 10.78645 |
| KDM5D | -1.17369 | 6.205486 | -4.50236 | 1.44E-05 | 0.000131 | 2.723462 |
| USP9Y | -1.12587 | 5.393411 | -4.05107 | 8.57E-05 | 0.00054 | 1.047025 |
| ANGPT2 | 1.047516 | 7.131025 | 6.211617 | 6.10E-09 | 2.93E-07 | 10.13557 |
| MS4A15 | -1.04069 | 8.09228 | -6.56022 | 1.06E-09 | 7.07E-08 | 11.81642 |
| ASPN | 1.012341 | 7.825212 | 5.786498 | 4.81E-08 | 1.45E-06 | 8.154738 |
| TXLNGY | -0.99498 | 5.76966 | -4.35978 | 2.57E-05 | 0.000208 | 2.179438 |
| OGN | 0.980267 | 8.393405 | 6.939686 | 1.51E-10 | 1.47E-08 | 13.69785 |
| MTND2P28 | -0.9705 | 9.569397 | -6.37197 | 2.75E-09 | 1.52E-07 | 10.90279 |
| CCDC80 | 0.960767 | 8.97339 | 5.802701 | 4.45E-08 | 1.37E-06 | 8.228744 |
| S100A12 | -0.94402 | 7.74032 | -4.30698 | 3.17E-05 | 0.000246 | 1.981263 |
| SELE | 0.938807 | 6.831061 | 3.90622 | 0.000148 | 0.000835 | 0.537977 |
| SAA2 | -0.92842 | 6.259591 | -3.8279 | 0.000197 | 0.001045 | 0.268854 |
| RGS1 | 0.908127 | 8.360392 | 6.352065 | 3.03E-09 | 1.63E-07 | 10.80696 |
| BPIFA1 | -0.90048 | 5.788915 | -5.61525 | 1.08E-07 | 2.71E-06 | 7.380176 |
| FCN3 | -0.90013 | 10.89822 | -5.62 | 1.06E-07 | 2.67E-06 | 7.401427 |
| TSHZ2 | 0.900116 | 7.529663 | 7.997264 | 5.17E-13 | 1.93E-10 | 19.17648 |
| PDGFD | 0.895883 | 6.316762 | 8.099938 | 2.94E-13 | 1.29E-10 | 19.72364 |
| CHIT1 | -0.87985 | 6.606748 | -5.36305 | 3.46E-07 | 6.86E-06 | 6.265668 |
| SCARNA4 | -0.87816 | 6.190379 | -6.12733 | 9.24E-09 | 4.09E-07 | 9.736561 |
| PDE1A | 0.861335 | 6.201581 | 7.953375 | 6.58E-13 | 2.17E-10 | 18.9433 |
| ENPP2 | 0.85411 | 9.908933 | 5.830679 | 3.89E-08 | 1.24E-06 | 8.356823 |
| SAA1 | -0.84849 | 4.195423 | -3.85748 | 0.000177 | 0.000962 | 0.369975 |
| HAS2 | 0.84224 | 7.935377 | 4.552814 | 1.17E-05 | 0.000111 | 2.919032 |
| SLCO4A1 | -0.84145 | 7.989127 | -5.66863 | 8.40E-08 | 2.24E-06 | 7.620075 |
| VIPR1 | -0.83967 | 9.647287 | -5.64208 | 9.52E-08 | 2.47E-06 | 7.500579 |
| FAP | 0.827636 | 6.177555 | 5.501466 | 1.83E-07 | 4.10E-06 | 6.873374 |
| IL13RA2 | 0.824648 | 4.246515 | 5.472199 | 2.10E-07 | 4.61E-06 | 6.744066 |
| IGF1 | 0.82181 | 7.386483 | 6.224165 | 5.73E-09 | 2.82E-07 | 10.19522 |
| MSMB | -0.82051 | 4.695612 | -3.4677 | 0.000705 | 0.002931 | -0.91178 |
| ZFY | -0.81535 | 5.3262 | -4.12927 | 6.35E-05 | 0.000426 | 1.327862 |
| RGS5 | 0.815261 | 9.47097 | 7.598997 | 4.54E-12 | 1.01E-09 | 17.07747 |
| SNORD114-2 | 0.810108 | 4.695526 | 6.156794 | 8.00E-09 | 3.66E-07 | 9.875702 |
| ANKRD36B | 0.809073 | 7.897858 | 8.288225 | 1.03E-13 | 5.40E-11 | 20.73289 |
| GEM | 0.804761 | 8.237639 | 7.372757 | 1.53E-11 | 2.67E-09 | 15.90335 |
| ABCB1 | 0.801351 | 6.420655 | 5.882428 | 3.04E-08 | 1.02E-06 | 8.594673 |
| S100A9 | -0.79733 | 8.815288 | -6.09487 | 1.08E-08 | 4.65E-07 | 9.583698 |
| WIF1 | 0.788911 | 10.51504 | 5.241293 | 6.02E-07 | 1.07E-05 | 5.739337 |
| ESM1 | 0.783306 | 6.425385 | 4.98128 | 1.90E-06 | 2.61E-05 | 4.642186 |
| ACE2 | 0.780918 | 6.008043 | 9.352317 | 2.52E-16 | 4.74E-13 | 26.55232 |
| CA1 | 0.774717 | 5.762094 | 6.48739 | 1.54E-09 | 9.51E-08 | 11.46133 |
| VTRNA1-1 | -0.77326 | 5.776522 | -3.83797 | 0.00019 | 0.001015 | 0.303217 |
| PAMR1 | 0.764656 | 6.770709 | 6.022956 | 1.54E-08 | 6.04E-07 | 9.246679 |
| ADRA1A | -0.758 | 6.288325 | -8.44905 | 4.21E-14 | 2.64E-11 | 21.60053 |
| RNASE2 | -0.75744 | 6.394706 | -5.2025 | 7.16E-07 | 1.21E-05 | 5.573304 |
| CFH | 0.749688 | 9.598893 | 7.444552 | 1.04E-11 | 1.98E-09 | 16.27441 |
| ABCC9 | 0.743924 | 8.543045 | 8.478373 | 3.58E-14 | 2.32E-11 | 21.75926 |
| ALAS2 | 0.737329 | 6.52961 | 7.371007 | 1.55E-11 | 2.67E-09 | 15.89432 |
| AREG | 0.736552 | 9.391231 | 4.429888 | 1.94E-05 | 0.000166 | 2.445304 |
| HMCN1 | 0.736545 | 8.703287 | 8.086105 | 3.17E-13 | 1.30E-10 | 19.6498 |
| FGF7 | 0.736298 | 6.468751 | 5.375967 | 3.27E-07 | 6.52E-06 | 6.321966 |
| ECM2 | 0.732586 | 7.202747 | 8.011297 | 4.79E-13 | 1.85E-10 | 19.25113 |
| MACC1 | 0.726211 | 8.119488 | 6.52283 | 1.28E-09 | 8.22E-08 | 11.63388 |
| PDE7B | 0.721248 | 6.918689 | 7.853377 | 1.14E-12 | 3.57E-10 | 18.4137 |
| SULF1 | 0.720749 | 7.914191 | 7.47666 | 8.78E-12 | 1.70E-09 | 16.44082 |
| ITGA2 | 0.720251 | 8.943964 | 5.122791 | 1.02E-06 | 1.58E-05 | 5.234687 |
| TDO2 | 0.716666 | 5.426882 | 3.504029 | 0.000623 | 0.002658 | -0.79705 |
| EPHA3 | 0.714902 | 6.712872 | 8.317693 | 8.77E-14 | 4.86E-11 | 20.8915 |
| ANK2 | 0.706142 | 6.325307 | 7.978626 | 5.73E-13 | 1.97E-10 | 19.07741 |
| BTNL9 | -0.70563 | 8.806049 | -5.74216 | 5.94E-08 | 1.72E-06 | 7.952857 |
| LRRN4 | -0.70473 | 8.642224 | -6.56974 | 1.01E-09 | 6.81E-08 | 11.86302 |
| NT5E | 0.703553 | 6.714603 | 9.272325 | 3.98E-16 | 6.28E-13 | 26.10928 |
| TMEM100 | -0.70257 | 9.091378 | -3.82284 | 0.000201 | 0.001059 | 0.251621 |
| MIR32 | 0.69188 | 6.469548 | 5.428433 | 2.57E-07 | 5.43E-06 | 6.551508 |
| LTBP1 | 0.690957 | 9.396545 | 8.718341 | 9.28E-15 | 8.73E-12 | 23.06385 |
| GBP5 | 0.690886 | 7.084181 | 5.751545 | 5.68E-08 | 1.66E-06 | 7.995504 |
| C5 | 0.690754 | 7.995711 | 6.945655 | 1.46E-10 | 1.44E-08 | 13.72785 |
| S100A8 | -0.6874 | 10.45302 | -4.49311 | 1.50E-05 | 0.000135 | 2.68774 |
| SERPINE2 | 0.685335 | 7.161031 | 5.125139 | 1.01E-06 | 1.57E-05 | 5.24461 |
| TM4SF18 | 0.683909 | 8.225171 | 5.098302 | 1.14E-06 | 1.72E-05 | 5.131352 |
| SNORD113-3 | 0.683819 | 4.670022 | 6.266866 | 4.64E-09 | 2.36E-07 | 10.39872 |
| AGBL1 | 0.68309 | 7.647256 | 5.404645 | 2.86E-07 | 5.92E-06 | 6.447259 |
| SNORA60 | -0.68227 | 8.281348 | -4.34103 | 2.77E-05 | 0.000221 | 2.108865 |
| INHBA | 0.681905 | 7.505076 | 6.448415 | 1.87E-09 | 1.10E-07 | 11.27214 |
| NR1D2 | 0.681617 | 8.291968 | 6.056813 | 1.31E-08 | 5.35E-07 | 9.405069 |
| SNORD113-4 | 0.681038 | 5.100194 | 5.183885 | 7.78E-07 | 1.29E-05 | 5.493906 |
| SEMA3D | 0.680698 | 7.258639 | 5.462341 | 2.20E-07 | 4.79E-06 | 6.70061 |
| CD69 | 0.679481 | 8.422208 | 5.302191 | 4.57E-07 | 8.59E-06 | 6.001614 |
| CA4 | -0.67392 | 7.607335 | -7.02959 | 9.42E-11 | 1.05E-08 | 14.15087 |
| ANKRD36 | 0.672835 | 9.294175 | 10.4918 | 3.46E-19 | 6.51E-15 | 32.92437 |
| KLHL4 | 0.672202 | 4.913949 | 7.733779 | 2.19E-12 | 6.09E-10 | 17.78344 |
| ADAMTS9 | 0.672161 | 9.177564 | 4.762915 | 4.87E-06 | 5.59E-05 | 3.750338 |
| TTN | 0.671248 | 8.233519 | 4.059742 | 8.29E-05 | 0.000525 | 1.077971 |
| LINC01140 | 0.67091 | 7.703867 | 6.841617 | 2.51E-10 | 2.24E-08 | 13.20678 |
| SNORD74 | -0.67036 | 8.801957 | -5.39002 | 3.06E-07 | 6.23E-06 | 6.383327 |
| BIRC3 | 0.665553 | 8.601254 | 4.711665 | 6.05E-06 | 6.61E-05 | 3.545089 |
| ZNF385D | 0.66386 | 6.351502 | 6.139271 | 8.72E-09 | 3.93E-07 | 9.79291 |
| HIVEP2 | 0.66347 | 8.501607 | 7.092074 | 6.79E-11 | 8.09E-09 | 14.46724 |
| ACADL | 0.663384 | 8.130423 | 5.571208 | 1.33E-07 | 3.22E-06 | 7.183234 |
| DCLK1 | 0.662097 | 6.440774 | 6.36744 | 2.81E-09 | 1.55E-07 | 10.88094 |
| BCHE | 0.661095 | 5.373564 | 5.078878 | 1.24E-06 | 1.84E-05 | 5.049629 |
| C1orf194 | -0.65955 | 7.142125 | -3.44259 | 0.000768 | 0.003143 | -0.9905 |
| BICC1 | 0.657825 | 7.263976 | 8.641808 | 1.43E-14 | 1.23E-11 | 22.64672 |
| RSPO3 | 0.65705 | 6.726434 | 7.581736 | 4.99E-12 | 1.08E-09 | 16.98739 |
| CCDC141 | 0.652745 | 7.712904 | 3.956706 | 0.000122 | 0.000722 | 0.713748 |
| PDE3A | 0.651707 | 7.644969 | 8.269346 | 1.15E-13 | 5.84E-11 | 20.63137 |
| FAM171B | 0.647104 | 7.373918 | 8.038066 | 4.13E-13 | 1.65E-10 | 19.39364 |
| ANKRD28 | 0.645281 | 8.706452 | 6.94535 | 1.46E-10 | 1.44E-08 | 13.72631 |
| LRRC32 | -0.64414 | 9.132249 | -5.90882 | 2.68E-08 | 9.26E-07 | 8.716461 |
| IFI44L | 0.642 | 8.333102 | 3.865144 | 0.000172 | 0.00094 | 0.396284 |
| CSF3R | -0.64169 | 8.734172 | -8.62091 | 1.61E-14 | 1.31E-11 | 22.53298 |
| SNORA7B | -0.63688 | 8.653963 | -3.74075 | 0.000271 | 0.001351 | -0.02549 |
| MEDAG | 0.636453 | 7.279124 | 3.742423 | 0.000269 | 0.001345 | -0.01989 |
| HIVEP1 | 0.636134 | 8.709696 | 9.449024 | 1.45E-16 | 3.35E-13 | 27.08889 |
| ITGB3 | 0.635418 | 8.234139 | 4.635432 | 8.32E-06 | 8.44E-05 | 3.242716 |
| HIF3A | -0.63334 | 7.799045 | -5.59552 | 1.18E-07 | 2.92E-06 | 7.291838 |
| LRRC17 | 0.631464 | 5.892835 | 5.57272 | 1.32E-07 | 3.20E-06 | 7.189979 |
| S100A3 | -0.63119 | 6.37617 | -4.9069 | 2.63E-06 | 3.39E-05 | 4.335288 |
| SLC9A3R2 | -0.62991 | 8.916826 | -9.5872 | 6.54E-17 | 2.46E-13 | 27.85723 |
| NKD1 | -0.62823 | 7.944209 | -8.13705 | 2.39E-13 | 1.10E-10 | 19.92197 |
| PLAC8 | -0.6272 | 8.765353 | -7.41277 | 1.24E-11 | 2.22E-09 | 16.10997 |
| TBX3 | -0.62705 | 8.173785 | -7.74612 | 2.04E-12 | 5.83E-10 | 17.84829 |
| ZNF521 | 0.626951 | 7.349083 | 7.112933 | 6.08E-11 | 7.48E-09 | 14.57314 |
| WEE1 | 0.625189 | 7.743633 | 5.352665 | 3.63E-07 | 7.11E-06 | 6.220477 |
| SLC7A2 | 0.623097 | 8.664614 | 6.425897 | 2.10E-09 | 1.22E-07 | 11.16309 |
| SLC6A4 | -0.62185 | 9.596232 | -3.00165 | 0.003201 | 0.010004 | -2.29322 |
| LILRA2 | -0.61896 | 5.80511 | -7.07193 | 7.54E-11 | 8.79E-09 | 14.36511 |
| CPA3 | 0.618013 | 9.05834 | 4.294287 | 3.33E-05 | 0.000256 | 1.933915 |
| GPR146 | -0.61706 | 7.505602 | -5.83103 | 3.89E-08 | 1.24E-06 | 8.358426 |
| XAF1 | 0.616784 | 9.272962 | 6.829173 | 2.68E-10 | 2.35E-08 | 13.1447 |
| MATN2 | 0.615838 | 7.174067 | 7.676983 | 2.98E-12 | 7.37E-10 | 17.48538 |
| PTPN13 | 0.614492 | 9.675208 | 6.96193 | 1.34E-10 | 1.38E-08 | 13.80969 |
| RASGRP1 | 0.614014 | 7.351627 | 6.273555 | 4.49E-09 | 2.30E-07 | 10.43066 |
| LCN2 | -0.61064 | 7.838411 | -4.63475 | 8.35E-06 | 8.46E-05 | 3.240024 |
| CXCL9 | 0.60994 | 7.141521 | 3.540397 | 0.000549 | 0.002399 | -0.68121 |
| FAM184A | 0.60846 | 7.331195 | 7.42415 | 1.16E-11 | 2.15E-09 | 16.16882 |
| JPX | 0.60827 | 5.871151 | 6.948752 | 1.44E-10 | 1.43E-08 | 13.74341 |
| HSPH1 | 0.601297 | 8.949564 | 4.841419 | 3.49E-06 | 4.28E-05 | 4.067766 |
| THY1 | 0.600427 | 7.684204 | 4.299916 | 3.26E-05 | 0.000251 | 1.954909 |
| RAMP2 | -0.59979 | 7.945581 | -4.05333 | 8.50E-05 | 0.000536 | 1.055082 |
| ANKRD22 | 0.598723 | 5.903374 | 3.994994 | 0.000106 | 0.000641 | 0.848236 |
| ITGB6 | 0.597914 | 9.55678 | 6.752436 | 3.98E-10 | 3.27E-08 | 12.76308 |
| SECISBP2L | 0.59438 | 9.780712 | 6.708446 | 4.99E-10 | 4.01E-08 | 12.54525 |
| ABCA8 | 0.594122 | 8.329531 | 5.70017 | 7.24E-08 | 2.01E-06 | 7.762508 |
| DLG2 | 0.593465 | 5.935421 | 10.09628 | 3.45E-18 | 1.62E-14 | 30.70236 |
| PTGS2 | 0.591938 | 8.413726 | 3.713624 | 0.000298 | 0.001462 | -0.11599 |
| ANKRD1 | 0.591019 | 9.677795 | 2.856782 | 0.00496 | 0.014279 | -2.6874 |
| LUC7L3 | 0.589696 | 8.725941 | 5.708807 | 6.95E-08 | 1.95E-06 | 7.801593 |
| EPHA4 | 0.588056 | 7.851987 | 7.100218 | 6.50E-11 | 7.89E-09 | 14.50857 |
| MME | 0.588016 | 9.296198 | 5.023929 | 1.58E-06 | 2.25E-05 | 4.819574 |
| CFAP69 | 0.585708 | 6.387105 | 6.375414 | 2.70E-09 | 1.50E-07 | 10.91935 |
