# Supplementary material for: Integrated bioinformatic analysis reveals the underlying molecular mechanism of and potential drugs for pulmonary arterial hypertension
Source: Aging (Albany NY). 2021 May 18;13(10):14234–57. doi: 10.18632/aging.203040 (PMC8202883; doi:10.18632/aging.203040)
Supplement: Supplementary Tables 3 to 9 [file aging-13-203040-s004.pdf]

SUPPLEMENTARY TABLES

Supplementary Table 3. The result of PPI.

Algorithm	Gene
MCC	FGF7, POSTN, LCN2, S100A8, CSF3R, SAA1, S100A12, S100A9, ITGA2, ITGB3, CD69, MME, FAP, ABCB1, ASPN, SERPINE2, HAS2, SFRP2, INHBA, BIRC3, CFH, OGN, HBB, ECM2, SULF1, PDGFD, WIF1, RGS5, RSPO3, RNASE2, SAA2
DMNC	MME, AREG, CCDC80, SAA2, PDGFD, CSF3R, ITGA2, ANGPT2, IFI44L, S100A9, FGF7, FAP, SELE, SERPINE2, BIRC3, CXCL9, C5, SFRP2, HAS2, ANKRD22, NT5E, RGS5, SULF1, ACE2, S100A8, SAA1, ECM2, MEDAG, ADRA1A, VCAM1, THY1, CD69, S100A12, LUC7L3, RAMP2, PTPN13, LRRC17, RSPO3, LCN2, CA4
MNC	IGF1, PTGS2, POSTN, VCAM1, THY1, SELE, CXCL9, ASPN, LCN2, ITGB3, INHBA, FGF7, NT5E, ANGPT2, S100A8, SFRP2, AREG, FAP, CD69, ITGA2, S100A12, OGN, EPHA3, CSF3R, SAA1, CFH, TTN, S100A9, SERPINE2, HBB, ABCB1, DCLK1, HAS2, WIF1, LTBP1, BCHE, ANK2, SULF1, ECM2, BIRC3, EPHA4
Degree	IGF1, PTGS2, POSTN, VCAM1, THY1, SELE, CXCL9, ASPN, LCN2, ITGB3, INHBA, FGF7, NT5E, ANGPT2, S100A8, FAP, SFRP2, CD69, AREG, CFH, TTN, ITGA2, S100A12, OGN, EPHA3, CSF3R, ABCB1, SAA1, SERPINE2, S100A9, HBB, DCLK1, HAS2, WIF1, LTBP1, BCHE, ANK2, SULF1, ECM2, EPHA4
EPC	IGF1,PTGS2,POSTN,VCAM1,THY1,SELE,CXCL9,ASPN,LCN2,ITGB3,INHBA,FGF7,NT5E,ANGPT2,S100A8,FAP,SFRP2,CD69,AREG,CFH,TTN,ITGA2,S100A12,OGN,EPHA3,CSF3R,ABCB1,SAA1,SERPINE2,S100A9,DCLK,1,HAS2,WIF1,LTBP1,BCHE,SULF1,ECM2,BIRC3,ESM1,MME
BottleNeck	IGF1,PTGS2,POSTN,THY1,CXCL9,LCN2,FGF7,NT5E,ANGPT2,FAP,CD69,AREG,CFH,TTN,ITGA2,CSF3R,ABCB1,S100A9,DCLK1,WIF1,LTBP1,BCHE,EPHA4,PAMR1,GEM,RSPO3,TBX3,RGS5,DLG2,CCDC80,PDGFD,CPA3,LRRC32,ADAMTS9,MEDAG,LRRC17,TDO2,ANKRD22,ABCC9,WEE1,XAF1,LUC7L3,RAMP2,TSHZ2,LILRA2,SLC7A2,HIF3A,MSMB
Eccentricity	IGF1,PTGS2,POSTN,VCAM1,THY1,SELE,CXCL9,ASPN,LCN2,ITGB3,INHBA,FGF7,NT5E,ANGPT2,S100A8,FAP,SFRP2,CD69,AREG,CFH,TTN,ITGA2,S100A12,OGN,EPHA3,CSF3R,ABCB1,SAA1,SERPINE2,S100A9,HBB,DCLK1,HAS2,WIF1,LTBP1,BCHE,ANK2,SULF1,ECM2,EPHA4,BIRC3,ESM1,PAMR1,GEM,MME,RGS1,RSPO3,TBX3,ENPP2,RGS5,RNASE2,MATN2,DLG2,HMCN1,ITGB6,CCDC80,CA1,PDGFD,ACE2,GBP5,CPA3,LRRC32,PDE1A,SLC6A4,ADAMTS9,ANKRD1,ADRA1A,CA4,PI15,MEDAG,LRRC17,ALAS2,TDO2,ANKRD22,SEMA3D,WEE1,IFI44L,XAF1,LUC7L3,SAA2,SLC9A3R2,ABCA8,RAMP2,C5,BPIFA1,HSPH1,PLAC8,CHIT1,IL13RA2,ZNF521,ANKRD28,FCN3,S100A3,SLC7A2,RASGRP1,HIVEP2,VIPR1,PTPN13,HIF3A,MSMB,SLCO4A1,
Closeness	IGF1,PTGS2,POSTN,VCAM1,THY1,SELE,CXCL9,ASPN,LCN2,ITGB3,INHBA,FGF7,NT5E,ANGPT2,S100A8,FAP,SFRP2,CD69,AREG,CFH,TTN,ITGA2,S100A12,OGN,EPHA3,CSF3R,ABCB1,SAA1,SERPINE2,S100A9,HBB,DCLK1,HAS2,WIF1,LTBP1,BCHE,ANK2,ECM2,EPHA4,BIRC3,
Radiality	IGF1,PTGS2,POSTN,VCAM1,THY1,SELE,CXCL9,ASPN,LCN2,ITGB3,INHBA,FGF7,NT5E,ANGPT2,S100A8,FAP,SFRP2,CD69,AREG,CFH,TTN,ITGA2,OGN,EPHA3,CSF3R,ABCB1,SAA1,SERPINE2,HBB,DCLK1,HAS2,WIF1,LTBP1,BCHE,ANK2,ECM2,EPHA4,BIRC3,ESM1,TBX3,
Betweenness	IGF1,PTGS2,POSTN,VCAM1,THY1,SELE,CXCL9,ASPN,LCN2,ITGB3,INHBA,FGF7,NT5E,ANGPT2,S100A8,FAP,SFRP2,CD69,CFH,TTN,S100A12,OGN,EPHA3,CSF3R,ABCB1,SAA1,SERPINE2,DCLK1,WIF1,ANK2,EPHA4,PAMR1,GEM,TBX3,RNASE2,PDGFD,MEDAG,ABCC9,LUC7L3,LILRA2,
Stress	IGF1,PTGS2,POSTN,VCAM1,THY1,SELE,CXCL9,ASPN,LCN2,ITGB3,INHBA,FGF7,NT5E,ANGPT2,S100A8,FAP,SFRP2,CD69,CFH,TTN,S100A12,OGN,EPHA3,CSF3R,ABCB1,SAA1,SERPINE2,HBB,DCLK1,WIF1,ANK2,EPHA4,ESM1,PAMR1,GEM,TBX3,RNASE2,PDGFD,ABCC9,LUC7L3,LILRA2,

Clustering, coefficient	SELE,CXCL9,FGF7,NT5E,ANGPT2,S100A8,SFRP2,AREG,ITGA2,S100A12,CSF3R,SAA1,SERPINE2,S100A9,HAS2,SULF1,ECM2,BIRC3,MME,ENPP2,RGS5,HMCN1,CCDC80,CA1,PDGFD,ACE2,SLC6A4,ADAMTS9,CA4,SEMA3D,IFI44L,SAA2,SOSTDC1,C5,CHIT1,IL13RA2,LILRA2,PTPN13,BPIFB1,ANKRD36B,

Supplementary Table 4. The information of five hub genes.

Gene symbol	Full name	Log (fold change)	P value
CSF3R	colony stimulating factor 3 receptor	-0.641687	$1.61E^{-14}$
NT5E	5'-nucleotidase ecto	0.703553	$3.98E^{-16}$
ANGPT2	angiopoietin 2	1.047516	$6.1E^{-9}$
FGF7	fibroblast growth factor 7	0.736298	$3.27E^{-7}$
CXCL9	C-X-C motif chemokine ligand 9	0.609940	0.000549

Supplementary Table 5. The top 20 GSVA terms of ANGPT2.

Pathways	LogFC	P.value	FDR
KEGG_ONE_CARBON_POOL_BY_FOLATE	0.243792192	5.86E-05	0.001317033
KEGG_PROTEIN_EXPORT	0.233239809	0.000238508	0.002194269
KEGG_VALINE_LEUCINE_AND_ISOLEUCINE_BIOSYNTHESIS	0.232604737	0.000427454	0.003292363
KEGG_SPLICEOSOME	0.231820688	1.50E-05	0.000552429
KEGG_NON_HOMOLOGOUS_END_JOINING	0.210725052	0.002509695	0.012152209
KEGG_PROPANOATE_METABOLISM	0.192878556	0.002170981	0.011568672
KEGG_RNA_DEGRADATION	0.189000949	0.000137356	0.001534455
KEGG_CELL_CYCLE	0.186071751	7.77E-06	0.000476692
KEGG_MISMATCH_REPAIR	0.185866007	0.001760087	0.010120498
KEGG_NUCLEOTIDE_EXCISION_REPAIR	0.184198544	0.00014177	0.001534455
KEGG_MATURITY_ONSET_DIABETES_OF_THE_YOUNG	-0.179973823	0.001329905	0.008288554
KEGG_AMINOACYL_TRNA_BIOSYNTHESIS	0.176774474	0.004419167	0.01805783
KEGG_BASAL_TRANSCRIPTION_FACTORS	0.170206927	0.000470546	0.003463222
KEGG_GLYCOSPHINGOLIPID_BIOSYNTHESIS_GLOBO_SERIES	-0.167989923	0.000584972	0.004139802
KEGG_CYSTEINE_AND_METHIONINE_METABOLISM	0.166307349	1.32E-05	0.000552429
KEGG_SULFUR_METABOLISM	-0.165247017	0.002200563	0.011568672
KEGG_ECM_RECEPTOR_INTERACTION	0.156670637	3.40E-05	0.001042607
KEGG_COMPLEMENT_AND_COAGULATION_CASCADES	0.152255983	4.24E-05	0.001113465
KEGG_UBIQUITIN_MEDIATED_PROTEOLYSIS	0.150071579	9.25E-05	0.001511601
KEGG_CIRCADIAN_RHYTHM_MAMMAL	0.148754719	0.006024413	0.023146939

Supplementary Table 6. The top 20 GSVA terms of CSF3R.

Pathways	LogFC	P.value	FDR
KEGG_PROPANOATE_METABOLISM	-0.295856535	1.43E-06	0.000131203
KEGG_GLYCOSPHINGOLIPID_BIOSYNTHESIS_GLOBO_SERIES	0.275953058	4.08E-09	7.50E-07
KEGG_VALINE_LEUCINE_AND_ISOLEUCINE_DEGRADATION	-0.268552435	6.01E-05	0.001381529
KEGG_STEROID_BIOSYNTHESIS	0.237527029	0.000184989	0.003403804
KEGG_BUTANOATE_METABOLISM	-0.226972724	9.60E-06	0.000353348
KEGG_MISMATCH_REPAIR	-0.212629125	0.000318776	0.003910318
KEGG_GLYCOSAMINOGLYCAN_DEGRADATION	0.207225325	7.50E-06	0.000344979
KEGG_FATTY_ACID_METABOLISM	-0.187955978	0.000315136	0.003910318
KEGG_NON_HOMOLOGOUS_END_JOINING	-0.18645673	0.007698082	0.03219198

KEGG_CELL_CYCLE	-0.169366017	5.27E-05	0.001381529
KEGG_DNA_REPLICATION	-0.165048486	0.002357046	0.015489158
KEGG_SPLICEOSOME	-0.162908395	0.002795828	0.01607601
KEGG_MATURITY_ONSET_DIABETES_OF_THE_YOUNG	0.157441906	0.005180597	0.025947799
KEGG_SULFUR_METABOLISM	0.156650672	0.003757833	0.02033651
KEGG_GALACTOSE_METABOLISM	0.156212812	0.000123689	0.002528749
KEGG_LYSINE_DEGRADATION	-0.155685152	0.000437974	0.005036704
KEGG_GLYCOSPHINGOLIPID_BIOSYNTHESIS_GANGLIO_SERIES	0.151678381	0.001026703	0.008586969
KEGG_BETA_ALANINE_METABOLISM	-0.145542379	0.000570707	0.00583389
KEGG_GLYCOSAMINOGLYCAN_BIOSYNTHESIS_CHONDROITIN_SULFATE	0.144237139	0.000886173	0.007764567
KEGG_HOMOLOGOUS_RECOMBINATION	-0.141959376	0.002459603	0.015605755

Supplementary Table 7. The top 20 GSVA terms of CXCL9.

Pathways	LogFC	P.value	FDR
KEGG_GRAFT_VERSUS_HOST_DISEASE	0.282957086	1.27E-06	2.93E-05
KEGG_ALLOGRAFT_REJECTION	0.28130979	1.62E-07	7.47E-06
KEGG_PRIMARY_IMMUNODEFICIENCY	0.274200636	1.34E-08	1.24E-06
KEGG_INTESTINAL_IMMUNE_NETWORK_FOR_IGA_PRODUCTION	0.273124844	5.38E-09	9.90E-07
KEGG_TYPE_I_DIABETES_MELLITUS	0.216729898	5.40E-06	0.000110495
KEGG_AUTOIMMUNE_THYROID_DISEASE	0.205178161	1.03E-06	2.71E-05
KEGG_VALINE_LEUCINE_AND_ISOLEUCINE_DEGRADATION	-0.194472279	0.00419016	0.027535338
KEGG_PROPANOATE_METABOLISM	-0.186295189	0.003106107	0.021167542
KEGG_ASTHMA	0.178999868	0.000488451	0.005617185
KEGG_ANTIGEN_PROCESSING_AND_PRESENTATION	0.178873662	1.78E-05	0.000297079
KEGG_T_CELL_RECEPTOR_SIGNALING_PATHWAY	0.16991138	2.46E-07	9.06E-06
KEGG_BUTANOATE_METABOLISM	-0.159434902	0.002278434	0.017467998
KEGG_VIRAL_MYOCARDITIS	0.152406694	7.50E-06	0.00013791
KEGG_FATTY_ACID_METABOLISM	-0.146538754	0.005412575	0.034341853
KEGG_SYSTEMIC_LUPUS_ERYTHEMATOSUS	0.144855573	0.000747094	0.007650551
KEGG_CHEMOKINE_SIGNALING_PATHWAY	0.141466051	1.23E-07	7.47E-06
KEGG_BETA_ALANINE_METABOLISM	-0.140925451	0.000866423	0.008390627
KEGG_CELL_ADHESION_MOLECULES_CAMS	0.140619537	6.53E-07	2.00E-05
KEGG_HEMATOPOIETIC_CELL_LINEAGE	0.137185897	4.99E-05	0.000765681
KEGG_LEISHMANIA_INFECTION	0.132412529	0.000748424	0.007650551

Supplementary Table 8. The top 20 GSVA terms of NT5E.

Pathways	LogFC	P.value	FDR
KEGG_VALINE_LEUCINE_AND_ISOLEUCINE_DEGRADATION	0.268767259	5.92E-05	0.000726073
KEGG_PROPANOATE_METABOLISM	0.26782988	1.49E-05	0.000355897
KEGG_NON_HOMOLOGOUS_END_JOINING	0.241074038	0.000507512	0.003735287
KEGG_MATURITY_ONSET_DIABETES_OF_THE_YOUNG	-0.238695958	1.55E-05	0.000355897
KEGG_MISMATCH_REPAIR	0.237927072	5.06E-05	0.000664519
KEGG_PROTEIN_EXPORT	0.210218429	0.000977788	0.005758475
KEGG_GLYCOSPHINGOLIPID_BIOSYNTHESIS_GLOBO_SERIES	-0.206021643	1.99E-05	0.000366165
KEGG_FATTY_ACID_METABOLISM	0.203952086	8.62E-05	0.000933061
KEGG_NICOTINATE_AND_NICOTINAMIDE_METABOLISM	0.203586763	2.54E-08	4.68E-06

KEGG_AMINOACYL_TRNA_BIOSYNTHESIS	0.202799915	0.001030024	0.005758475
KEGG_RENIN_ANGIOTENSIN_SYSTEM	0.189854677	3.03E-06	0.000139364
KEGG_SPLICEOSOME	0.183352551	0.000725563	0.004450122
KEGG_LYSINE_DEGRADATION	0.182695153	3.14E-05	0.000480322
KEGG_BUTANOATE_METABOLISM	0.177215104	0.000656871	0.004167734
KEGG_SULFUR_METABOLISM	-0.176625112	0.00103277	0.005758475
KEGG_BASAL_TRANSCRIPTION_FACTORS	0.167026339	0.000605049	0.003976034
KEGG_COLORECTAL_CANCER	0.16453329	8.69E-08	8.00E-06
KEGG_NEUROACTIVE_LIGAND_RECEPTOR_INTERACTION	-0.164283705	8.32E-05	0.000933061
KEGG_OLFACTORY_TRANSDUCTION	-0.160357734	0.000263719	0.002426211
KEGG_RNA_DEGRADATION	0.159304048	0.001426328	0.007718949

Supplementary Table 9. The top 20 GSVA terms of FGF7.

Pathways	LogFC	P.value	FDR
KEGG_PROTEIN_EXPORT	0.387193054	1.47E-10	1.35E-08
KEGG_NON_HOMOLOGOUS_END_JOINING	0.350637284	2.03E-07	1.78E-06
KEGG_AMINOACYL_TRNA_BIOSYNTHESIS	0.341186071	9.03E-09	1.85E-07
KEGG_ONE_CARBON_POOL_BY_FOLATE	0.327332488	3.01E-08	4.61E-07
KEGG_MISMATCH_REPAIR	0.31217779	5.10E-08	5.87E-07
KEGG_SPLICEOSOME	0.292664541	2.25E-08	3.76E-07
KEGG_VALINE_LEUCINE_AND_ISOLEUCINE_BIOSYNTHESIS	0.291787649	7.32E-06	3.74E-05
KEGG_CELL_CYCLE	0.274431256	4.17E-12	7.68E-10
KEGG_PROTEASOME	0.267658925	1.61E-05	7.41E-05
KEGG_RNA_DEGRADATION	0.265276504	3.62E-08	5.12E-07
KEGG_NOD_LIKE_RECEPTOR_SIGNALING_PATHWAY	0.263666936	1.68E-09	7.74E-08
KEGG_SULFUR_METABOLISM	-0.26215574	5.76E-07	3.99E-06
KEGG_NUCLEOTIDE_EXCISION_REPAIR	0.24581461	2.03E-07	1.78E-06
KEGG_BASAL_TRANSCRIPTION_FACTORS	0.245156814	2.30E-07	1.92E-06
KEGG_CITRATE_CYCLE_TCA_CYCLE	0.236617885	3.59E-05	0.000157218
KEGG_DNA_REPLICATION	0.235961202	9.30E-06	4.60E-05
KEGG_AMINO_SUGAR_AND_NUCLEOTIDE_SUGAR_METABOLISM	0.2267432	2.36E-06	1.31E-05
KEGG_PROPANOATE_METABOLISM	0.221093362	0.000404135	0.001352016
KEGG_HOMOLOGOUS_RECOMBINATION	0.218482259	1.75E-06	1.08E-05
KEGG_TERPENOID_BACKBONE_BIOSYNTHESIS	0.211187654	0.00022772	0.00080578
